# Geo-Referenced, Abundance Calibrated Ocean Distribution of Chinook Salmon (*Oncorhynchus tshawytscha*) Stocks across the West Coast of North America

**DOI:** 10.1371/journal.pone.0131276

**Published:** 2015-07-22

**Authors:** M. Renee Bellinger, Michael A. Banks, Sarah J. Bates, Eric D. Crandall, John Carlos Garza, Gil Sylvia, Peter W. Lawson

**Affiliations:** 1 Coastal Oregon Marine Experiment Station, Hatfield Marine Science Center, Department of Fisheries and Wildlife, Oregon State University, Newport, Oregon, United States of America; 2 California Salmon Council, Oakland, California, United States of America; 3 Southwest Fisheries Science Center, National Marine Fisheries Service, National Oceanographic and Atmospheric Administration, Santa Cruz, California, United States of America; 4 Institute of Marine Sciences, University of California Santa Cruz, Santa Cruz, California, United States of America; 5 Coastal Oregon Marine Experiment Station, Hatfield Marine Science Center, Department of Applied Economics, Oregon State University, Newport, Oregon, United States of America; 6 Northwest Fisheries Science Center, National Marine Fisheries Service, National Oceanographic and Atmospheric Administration, Newport, Oregon, United States of America; Institute of Marine Research, NORWAY

## Abstract

Understanding seasonal migration and localized persistence of populations is critical for effective species harvest and conservation management. Pacific salmon (genus *Oncorhynchus*) forecasting models predict stock composition, abundance, and distribution during annual assessments of proposed fisheries impacts. Most models, however, fail to account for the influence of biophysical factors on year-to-year fluctuations in migratory distributions and stock-specific survival. In this study, the ocean distribution and relative abundance of Chinook salmon (*O*. *tshawytscha*) stocks encountered in the California Current large marine ecosystem, U.S.A were inferred using catch-per-unit effort (CPUE) fisheries and genetic stock identification data. In contrast to stock distributions estimated through coded-wire-tag recoveries (typically limited to hatchery salmon), stock-specific CPUE provides information for both wild and hatchery fish. Furthermore, in contrast to stock composition results, the stock-specific CPUE metric is independent of other stocks and is easily interpreted over multiple temporal or spatial scales. Tests for correlations between stock-specific CPUE and stock composition estimates revealed these measures diverged once proportional contributions of locally rare stocks were excluded from data sets. A novel aspect of this study was collection of data both in areas closed to commercial fisheries and during normal, open commercial fisheries. Because fishing fleet efficiency influences catch rates, we tested whether CPUE differed between closed area (non-retention) and open area (retention) data sets. A weak effect was indicated for some, but not all, analyzed cases. Novel visualizations produced from stock-specific CPUE-based ocean abundance facilitates consideration of how highly refined, spatial and genetic information could be incorporated in ocean fisheries management systems and for investigations of biogeographic factors that influence migratory distributions of fish.

## Introduction

Ocean fishery management depends on understanding fish stock abundance and migratory patterns of movement to meet the dual objectives of conservation and harvest [1]. Pacific salmon (*Oncorhynchus* spp.) provide an interesting case study because they are highly migratory, variable in abundance [2,3], and have high ecological, cultural, and economic importance. Most species rear in freshwater, migrate to the ocean where they grow, and return to freshwater to spawn and die. They enrich freshwater habitat by transferring nutrients from the ocean, providing food for a wide variety of animals and fertilizing the surrounding vegetation. Genetic stock structure in salmon arises from fidelity to their natal streams and the timing of their breeding readiness, two traits that permit adaptation to the local environment [4–6]. More than 100 genetically differentiated stocks of Chinook salmon (*O*. *tshawytscha*) originate from the west coast of North America from Alaska to California [7–9]. During ocean migration, salmon form mixed stock aggregations that are often subject to fishing pressure. Salmon fishery management therefore requires knowledge of complex stock-specific life history patterns in concert with annual variation in stock-cohort abundance. Knowledge of seasonal migratory patterns and localized persistence of populations in the ocean are currently dependent on coarse-scale historical data. In this study, we explore the use of fine-scale sampling of Chinook salmon catch and effort in ocean fisheries, along with genetic stock identification (GSI), to describe stock-specific local abundance and migration patterns.

The Pacific Fishery Management Council (PFMC) develops management measures for Chinook salmon ocean fisheries in the southern portion of the California Current large marine ecosystem, United States of America (USA) [10]. A single-season modeling tool called the “Fishery Regulation Assessment Model” (FRAM) is used by the PFMC to predict cohort-based stock abundance and time and area stock compositions [11]. Using those modeled data, fishery harvest scenarios are analyzed to assess impacts to stocks, with the end goal of maximizing harvest while meeting conservation targets. This model relies heavily on mark and recapture data from mostly hatchery fish implanted with coded-wire-tags (CWT) that indicate source stock and cohort year [12,13]. Fish are sampled when landed at port or on return to hatcheries. The CWT recoveries are expanded by sampling (usually about 20%) and marking rates (usually about 5%) to estimate the number of tagged and untagged fish from each mark group in the modeled fishery [13]. CWT release groups are often used as “indicator stocks” for unmarked natural production. The FRAM model assumes (see model documentation for a full list) that sampling for CWTs is random, that CWT fish accurately represent the modeled stock, and that stock distributions and migratory timings are constant from year to year. However, mark selective fisheries implemented in recent years require the release of some fish. Furthermore, evidence is accumulating that some hatchery fish are less fit than their wild stock counterparts [14,15], and spatial and temporal fluctuations in marine environmental conditions influence stock distribution and survival [16–18]. Perhaps the most troubling aspect of modeling fisheries with CWT data is the delay in compiling all the recovery data required to reconstruct complete cohorts and estimate stock composition in fisheries. The precision and usefulness of fisheries management models would likely be increased by using data that more precisely estimate fishery stock composition, provide additional information about relative stock abundance, and are available in a timelier manner.

Genetic stock identification (GSI) [19–21] and CWTs are two tools that have proven useful for identifying individual fish to stock of origin and to model the proportion of stocks present in a fishery sample. GSI compares genetic profiles of samples with unknown stock origins against a reference “baseline” database of genotypes from individuals with known origins [19,20]. One advantage GSI has over CWTs is that all salmon carry a genetic profile and can potentially be assigned to a stock of origin. Thus, GSI estimates are not biased by expansion factors inherent to proportional tagging programs, which most CWT programs are, and even small sample sizes can be data rich. Another difference is that tissue samples for GSI can be obtained non-lethally, whereas CWT recovery requires removal of the fish’s snout. For these reasons, fisheries managers in Canada [22,23] and Alaska [24] have implemented in-season GSI sampling or test fisheries that guide implementation of stock-specific exploitation targets. These management measures have resulted in concomitant benefits of greater fishing opportunity and strengthened conservation for stocks of concern. Despite these successes, and the potential for GSI to improve salmon fisheries management [25,26], incorporation of GSI into marine harvest management in mainland US waters is limited.

Chinook and coho salmon (O. *kisutch*) are the two predominant salmon species encountered in salmon fisheries of the California Current. Both have a southern spawning distribution [27] and use the cool, upwelled water in the coastal shelf as a migratory corridor and feeding ground [28,29]. Harvest of coho salmon has been severely restricted or completely closed off the coasts of the U.S. states of Oregon (OR) and California (CA) over the past two decades because of conservation concerns [3]. Although the Chinook salmon fishery has persisted, the failure of some stocks to meet conservation targets in recent years has resulted in large-scale time and area fishery closures. The salmon fishery would benefit from techniques that increase accuracy and spatial resolution of fisheries stock distribution forecasts and provide for finer-scale control of harvest impacts.

Concern for salmon conservation and a sustainable fishery led commercial salmon fishermen, fisheries managers, and scientists in OR, CA, and Washington states to band together to develop novel solutions to issues facing salmon management. Together we utilized newly developed genetic resources [8,30] and geo-referenced catch and fishing effort data to elucidate fine-scale patterns of relative abundance and distribution for Chinook salmon stocks encountered in the California Current ecosystem during the year 2010. Most GSI studies report only stock composition data. In this study, we use genetic stock identifications with high-resolution fisheries catch and effort data to calculate stock-specific catch per unit effort (SSCPUE). We then assessed correlations between SSCPUE and stock composition results to identify conditions that lead to discordance between these two measures and identify situations where each is more appropriate. The at-sea catch and effort data were collected by fishermen using one of two sampling techniques: “retention” (open commercial fishery, fish retained for sale) or non-retention (areas closed to commercial fishing but open to catch and release sampling). To determine if sampling technique had an effect on CPUE, we analyzed catch rates for time-area strata that had both types of sampling conducted within short time periods.

Novel assessments and visualizations of stock-specific ocean distribution patterns facilitate the consideration of how highly refined spatial information might be incorporated into ocean salmon fishery management and used to better understand fish migration. The methods developed here are broadly applicable for measuring the migratory distribution and abundance data of any group of populations for which stock-origin and CPUE data are available.

## Methods

### At-sea data collection and sampling

Salmon troll fishermen in possession of active, state-issued commercial fishing licenses (issued by California or Oregon Departments of Fish and Wildlife) collected all Chinook salmon fin-clip samples used in this study. These fin-clips were collected from fish caught during an open fishery and retained as part of commercial fisheries harvest (“retention fishery”) or from fish caught and released in areas closed to commercial fishing but open to non-retention sampling (“non-retention sampling”) as authorized by the Pacific Fishery Management Council and other permits, described in detail below. For samples collected during the open fishery, after a fish retained for commercial sale was terminated, the fishermen removed from each fish a small fin-clip for this study. No additional permits were required for obtaining fin-clip samples from commercially harvested fish. For samples collected in areas closed to commercial fisheries but open to non-retention sampling, commercially licensed salmon fishermen used the same troll method to catch fish, but the fish were sampled and released alive [31]. Each fish was brought up to the side of the boat in a soft net (with no knots), handled as gently and as quickly as possible, and released after obtaining a small (typically < 1 cm x 1 cm) fin-clip. This non-retention sampling activity was permitted by: National Marine Fisheries Service Scientific Research Permit, Scientific Collecting Permits issued by the OR Department of Fish and Wildlife and the CA Department of Fish and Game (now Wildlife), and a letter from the International Pacific Halibut Commission. For the non-retention fishery one author, Peter W. Lawson, National Marine Fisheries Service and one non-author, Churchill Grimes, National Marine Fisheries, were in possessions of the permits. The permits specifically covered commercially licensed fishermen participating in this study to obtain fin clippings from the fish that were sampled from the non-retention fishery. The non-retention fin-clippings were taken for the purpose of the research described in this study, additional research, and for the development of fisheries management applications using genetic stock identification. The non-retention sampling impacts were allocated as "GSI Sampling Impacts" by the Pacific Fishery Management Council during the 2010 salmon season setting process.

**Fig 1:**
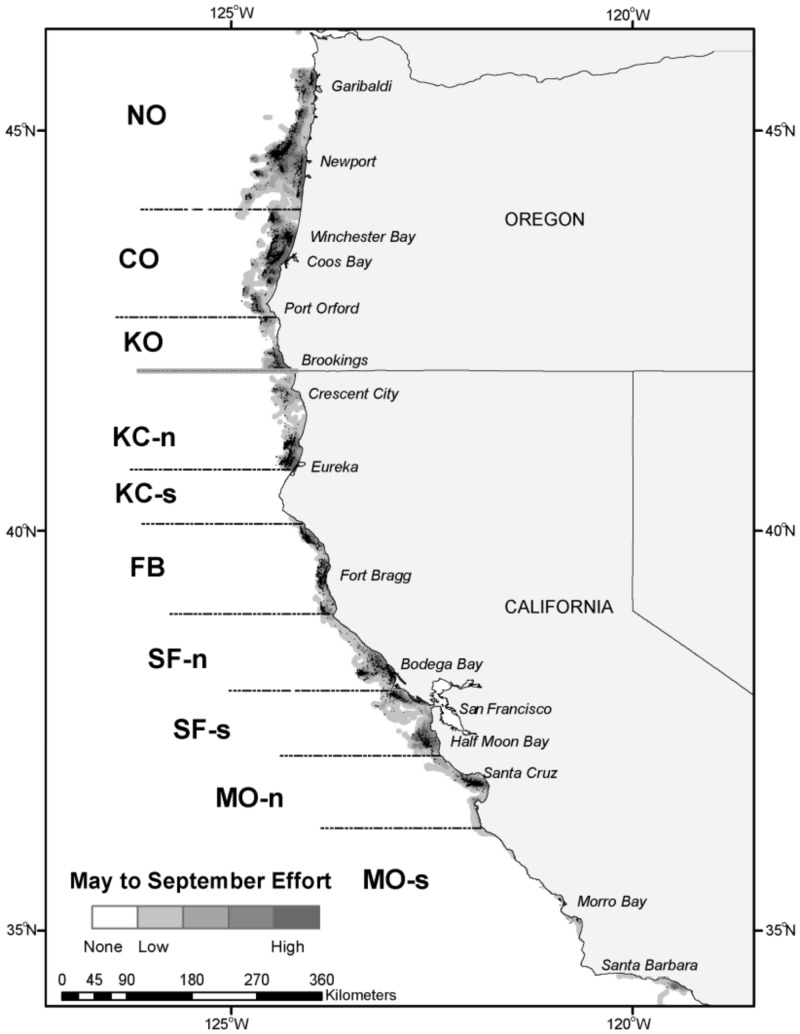
Troll fishing effort and Chinook salmon catch locations. Catch locations (n = 9,584) are shown as black dots while vessel-days effort (n = 2651) are conveyed as shaded contours. Fishing vessels locations were logged by GPS units in five-minute intervals. Regional boundaries with area abbreviations are: Cape Falcon to Florence south jetty, North Oregon Coast (NO, latitude (lat) 45.767° to 44.015°N); Florence south Jetty to Humbug Mountain, Central OR Coast (CO, 44.015° to 42.667°N); Humbug Mountain to CA/OR border, Klamath Zone OR (KO, 42.667° to 42.000°N); CA/OR border to Humboldt south jetty, Klamath Zone California-north (KC-n, 42.000° to 40.765°N, with no sampling permitted in the KC-s between 40.765°N to 40.083°N); Horse Mountain to Point Arena, Fort Bragg (FB, 40.083° to 38.958°); Point Arena to Point Reyes, San Francisco north (SF-n, 38.958° to 37.996°N); Point Reyes to Pigeon Point, San Francisco south (SF-s, 37.996° to 37.183°N); Pigeon Point to Point Sur, Monterey north (MO-n, 37.183° to 36.300°N) and Point Sur to Mexican Border, Monterey south (MO-s, 36.300° to 32.584°N).

At-sea data collection and biological sampling were conducted by commercial troll salmon fishermen in coastal waters of the California Current large marine ecosystem from Cape Falcon, OR (latitude (lat) 45.77° North (N)) southward to near the CA Channel Islands (lat 32.53°N), bounded by approximate longitudes 125.00° West to 120.000° West. A stratified sampling plan was implemented with the objective of collecting tissue samples from 200 legal-sized Chinook salmon (typically three years of age or older) per week (~ 800 per month) from six of seven fisheries management zones managed by the PFMC (Fig 1): North Oregon Coast (NO), lat 45.767° to 44.015°N; Central Oregon Coast (CO), lat 44.015° to 42.667°N; Klamath Zone Oregon (KO), lat 42.667° to 42.000° N; Klamath Zone California North (KC-n), lat 42.000° to 40.765°N (with no sampling permitted in the KC-south 40.765° to lat 40.083° N); Fort Bragg (FB), lat 40.083° to 38.958°N; and Monterey (MO), lat 37.183° to 32.584°N. The seventh zone, San Francisco (SF), lat 38.958° to 37.183°N, was divided at the Point Reyes peninsula (37.996°N) into north (SF-n) and south (SF-s) areas with the 200 sample size objective for each area. The MO zone was sampled as a single unit, but data were divided at Point Sur (36.300°N) into north (MO-n) and south (MO-s) areas for analysis purposes. Accordingly, results and data are presented for a total of nine different area strata.

For PMFC managed fisheries south of Cape Falcon, the forecasted low-abundance of CA Central Valley fall stock sparked conservation concerns. Consequently, the 2010 CA commercial fishery was mostly closed [10]. However, to enable broader sampling, the PFMC devised fishing regulations to allow for scientific impacts from non-retention sampling (up to 200 fish legal- and sub-legal sized) in time-area strata closed to commercial fisheries. A maximum of five vessels, required to remain within their designated area, were allowed to expend up to 15 vessel days of effort (total) per closed week-area stratum. Retention fisheries, by contrast, allowed for fishermen to fish (and sample) in whichever open sampling area they chose, and no limits were applied to the number of commercial fishing vessels allowed per time-area stratum (both sampling and non-participating fishing vessels). In CA, the SF and MO areas were only open to retention fisheries for eight days, July 1–4 and 8–11. The FB area was open during those days, plus July 15–29 and all of August. The OR fishery was open from May through August, except in the KO area, which was closed for the month of June. All areas from Cape Falcon to the US/Mexico international boundary were closed during September. Shore-based fleet managers in OR and CA coordinated vessel-days effort on a daily or weekly basis to actively manage progress towards sampling goals.

Fishermen collected a small fin clip for genetic analysis and recorded fish lengths at time of capture. Retention size limits differed slightly between OR and CA (28 and 27 inches total length, respectively). Fish with missing length data and sampled during non-retention fisheries were estimated as legal or sub-legal sized based on the proportion of known legal-sized to total fish sampled in the same month-area stratum. Catch locations and times were electronically logged by manually marking waypoints on a hand-held Global Positioning System (GPS) unit when fish were landed on the vessel deck. The same units were programmed to record, in five minute intervals, fishing effort (date, time, lat and longitude) for CPUE modeling.

### Comparison to commercial fishery

To provide context for this study we compared numbers of fish landed, vessel-days of fishing effort, and numbers of participating fishermen to the overall 2010 commercial fishery. The commercial fishery data were obtained from the PFMC’s Salmon Document Library: Historical Data of Ocean Salmon Fisheries “Blue Book” Appendix A, Ocean Salmon Fishery Effort and Landing, and Appendix D, Economic Data (available from http://www.pcouncil.org/salmon/background/document-library/historical-data-of-ocean-salmon-fisheries/). A simplifying assumption was that all PFMC data reported for ports Astoria and Tillamook were from fisheries conducted north or south of Cape Falcon, respectively. The OR September and October terminal fisheries catch and effort data were excluded from analysis.

### Statistical modeling of CPUE

#### Model selection and modeling variability in CPUE

Time and area variability in mean CPUE, defined as legal-sized catch per vessel-day of fishing effort, was statistically modeled with associated error using Generalized Linear Models (GLMs). The following model terms were considered: sample area (“area”), time-period (“time”, at week or month intervals), fishermen effect (a measure of individual fisherman power), and fishery sampling technique (retention or non-retention). Two types of GLMs, Poisson and log-linear negative binomial, were initially considered. CPUE data were overdispersed (likelihood ratio test Chi-square test statistic for overdispersion = 7400.98, p-value = < 2.2 e -16), and the negative binomial model was better supported than the Poisson model (Vuong non-nested hypothesis test statistic -18.49, p-value = 1.2 e -76 [32]). Therefore, log-linear negative binomial models were selected for modeling CPUE variability and to assess the predictive power of exogenous variables on fish catch.

Model performance was assessed using delta Akaike Information Criteria (ΔAIC) between a model with no terms and alternative models with terms. Individual terms were also tested for significant effects using an ANOVA with a significance cutoff of p < 0.05. The strength of each term’s effect was evaluated by the relative amount of decrease in residual deviance that resulted from that term’s inclusion. Only vessel-days (and catch) having GPS track log records and at least 85% of fishing effort expended in a single sampling area during a single day were included in GLM data sets. The CA July retention and non-retention data were combined for the month-area model. Week-area combinations having zero catch for all sample days (n = 14 days total within nine of 168 week-area combinations) were excluded because that pattern of data results in null values in the maximum likelihood estimator (quasi-complete separation problem [33]). For similar reasons, the terms “time” and “area” could not be modeled with the term “fishermen effect”. Analyses were performed in R version 2.15.2 with packages foreign (v 0.8–54), mass (v 7.3–23), car (v 2.0–18), lattice (0.20–23) and pscl (1.04.4).

#### Effect of non-retention and retention sampling technique on CPUE

We investigated the potential impact of sampling technique on fish catchability using two approaches. First, GLMs were used to statistically test for differences in CPUE between retention and non-retention fisheries. Five area-specific GLMs were run, each using data collected over approximately comparable time-periods. In four of the five area analyses, retention fisheries data collected July 1–4 and 8–11 were compared to non-retention fisheries data collected July 13–28 (areas SF-n, SF-s, MO-n, MO-s). The fifth area model, for FB, the June/non-retention was compared to July/retention fishery. Statistical significance was evaluated using an uncorrected p-value of < 0.05. For the second approach, a Chi-square test was used to evaluate whether retention and non-retention fisheries differed in the proportion of “successful” (at least one fish caught) versus “unsuccessful” (zero fish caught) fishing days. The data were partitioned by time period for this analysis: FB June/non-retention fishery were compared to July/retention fishery, and the SF- and MO early-July retention data were compared to the non-retention fishery data collected over the latter half of the month.

#### Effect of fisherman on CPUE

Individual fisherman skill and vessel efficiency is expected to vary across the fishing fleet, but measuring these effects on CPUE is confounded by inherent limitations of the study design. Individual fishermen sampled on an intermittent basis, typically in a single area. Because fish abundance varies over time and space, an individual fisherman’s catch rate cannot be specifically attributed to their prowess. In spite of these study design limitations, we evaluated the effect of individual fisherman performance on CPUE, because our results provide a rough idea of between-fisherman catch success, regardless of the cause. Model results may help guide future study design.

### Genetic stock identification

#### Oregon—microsatellites

Genomic DNA was extracted from fin-clips using silica-fiber Pall-plates [34] and arrayed into 384 well plates for genotyping. Polymerase chain reaction (PCR) was used to amplify 13 microsatellite loci standardized as part of an international baseline for Chinook salmon [8,9]. This baseline (v3.0) contains genotypes from over 30,000 Chinook salmon from 233 populations ranging from CA to Alaska, USA (S1 Appendix). Forward primers were fluorescently labeled and PCR products visualized using an Applied Biosystems model 3730xl Genetic Analyzer. GeneMapper software was used to assign standardized allele calls. Fish with identical or nearly identical genotypes (> 90% similarity) were identified using Microsatellite Toolkit [35] and excluded from analysis. Only fish that provided useful data at 7 or more loci were included in the final genetic data set.

#### California—single nucleotide polymorphisms

Samples collected off the coast of CA were genotyped using a panel of 96 single nucleotide polymorphism (SNP) markers [36] and the associated genetic baseline designed specifically for use in estimating stock composition in PFMC-managed fisheries [30]. SNP markers are both cheaper and faster to assay than microsatellites and have lower genotyping error and missing data rates. Genomic DNA was extracted from fin clips using DNEasy 96 filter kits on a BioRobot 3000 (QIAGEN Inc.) after digestion in proteinase K. A preliminary PCR was performed with primers for all 96 SNP loci, followed by individual locus PCRs performed on 96.96 Dynamic Genotyping Arrays (Fluidigm Corporation). Results were visualized using the EP1 instrumentation (Fluidigm) according to manufacturer’s protocols. Genotypes were scored with Fluidigm SNP Genotyping analysis software and identical or nearly identical genotypes were identified and filtered as detailed for microsatellites.

The SNP baseline database includes 68 populations that represent > 99% of all fish found in ocean fisheries off CA and OR [36]. The sampling of CA Chinook salmon populations is denser in the SNP than the microsatellite baseline. Chinook and coho salmon are sister species and are occasionally misidentified in the field. Thus, coho salmon genotypes for the 96 SNP markers were added to the baseline to identify and exclude that species of fish. Fish genotypes missing more than 20 loci or which had individual heterozygosities less than 0.16 or greater than 0.56, to correct for allelic dropout and contamination respectively, were removed from the final dataset.

#### Mixed stock fishery analysis

The program gsi_sim, which uses both genotype frequencies and mixture proportions when estimating the origin of individuals (available at http: http://swfsc.noaa.gov/textblock.aspx?Division=FED&ParentMenuId=54&id=12964) [37,38], was used for mixed stock analysis and individual assignments. A sliding-window approach was used as follows to represent the proportion of each reporting unit in the Bayesian prior. Genotype data were partitioned into weekly strata for each fishery management zone and each week’s data were then analyzed in the context of genotype frequencies observed in the weeks immediately before and after the focal week. Individual assignments were then collated into monthly stock proportions. GSI techniques generally assign all fish of unknown origin to a stock represented in the baseline. Here, we implemented a novel maximum likelihood method in gsi_sim and described by Clemento et al. [30] to evaluate whether fish may actually have originated from a stock/reporting group not represented in the baseline.

For GSI applications, populations were aggregated into ‘reporting groups’ consistent with Seeb et al. [8], with one exception. Here, the CA Central Valley spring stock from the Feather River was placed into the CA Central Valley fall reporting group because of known hybridization between these stocks [21,39]. After mixed stock fishery analysis was performed, higher-level regional groupings of Alaska, British Columbia, Canada (two groups: Vancouver Island / mainland and Fraser River basin) and Puget Sound stocks were used to reduce the total number of reporting groups. At the regional grouping levels used in this study, almost all reporting units are easily resolved with both baselines [8,30]. Known exceptions for the microsatellite baseline are low power to correctly assign fish to Deschutes fall [8,40] and some Columbia River (e.g., Snake River fall, Lower Columbia River spring [40]) stocks.

### Comparison between stock origins identified by GSI and CWT

The power of the microsatellite baseline to accurately assign individuals to source populations was empirically tested by comparing GSI results to stock identifications for fish with CWTs recovered during commercial fishery dockside sampling in OR. The GSI retention-sampled fish were labeled with physical barcodes (by fishermen) to enable cross-referencing by port samplers. Low confidence assignments (individual posterior probabilities of assignment < 0.90) and fish from stocks reared or released out-of-basin were excluded from CWT-GSI comparisons. Using the SNP baseline, a similar comparison to CWT data in CA was performed by Clemento et al. [30], but on a separate set of fishery samples.

### Stock richness, distribution and CPUE-based abundance patterns

Stock distribution, abundance and richness (the number of reporting groups present in a sample) for month-area strata were inferred by partitioning CPUE-based measures of abundance into stock-specific contribution estimates from GSI results (stock-specific catch per unit effort, SSCPUE). Here, CPUE was measured as the sum of legal-sized fish encounters (sampled and unsampled fish) divided by the sum of days fished per month-area stratum. Thus, SSCPUE for stock i in stratum j would be calculated as:

$$\text{SSCPUE }={\text{ stock}}_{\text{i}}{\text{stratum}}_{\text{j}}\times \left(\text{n legal}-\text{sized fish encounters}÷\text{n vessel}-\text{days effort }\right){\text{stratum}}_{\text{j}}$$

The resultant values represent the number (usually a fraction) of fish from each stock that fishermen would, on average, encounter per vessel-day fishing effort in a given stratum. This method accounts for unsampled fish and those that did not meet genotyping or GSI assignment criteria. If a vessel crossed over an area boundary during a single day, the effort was assigned in proportion to the amount of GPS-recorded time spent in each area and catch was allocated to the area where the fish was caught. Reporting groups that contributed to three or fewer strata were excluded to minimize the numbers of stocks in figures. Confidence intervals for individual SSCPUE values were not included, but overall sampling error can be inferred from CPUE modeling results. Simplifying assumptions were that GSI stock composition estimates were accurate, CPUE was unaffected by sampling technique, and that CPUE was proportional to abundance. Results for all stocks and strata are graphically presented with bar graphs in a “small multiples” (*sensu* Tufte [41]) format, with each element combining overall effort and log-transformed SSCPUEs for the month-area stratum. For a sub-set of stocks, distribution patterns are also presented as filled log-CPUE contour plots generated in SigmaPlot v11. Breaks in sampling coverage were not incorporated into contour plots because imperfect sampling coverage results in numerous breaks, depending on the time-area scale, and choosing which sections to mask is subjective. The time-frame “month” was selected for SSCPUE analyses because that is the interval used in PFMC fisheries management.

### Comparisons between stock composition and SSCPUE measures

Discord in the relationship between stock composition and corresponding values of SSCPUE is expected to occur because, for a target stock with constant abundance in a given area, a change in any other stocks’ local abundance will affect that stock’s proportion of the total catch composition but not SSCPUE results. Strengths of associations between SSCPUE and stock composition values were examined by calculating the non-parametric Kendall’s tau rank correlation coefficient (τ). This test evaluates the similarity of the orderings of the data ranked by each of the quantities and tests the data set against the null hypothesis of τ = 0 with a two-sided p-value of ≤ 0.05. Correlations between stock composition and SSCPUE values were first assessed using all pairs of non-zero data (“full data set”) from month-area strata (retention and non-retention combined). Then, correlations were re-evaluated after considering only data points above a range of threshold values of percent stock composition (“threshold data set”), iterating to find the interval at which Kendall’s τ correlations were reduced to non-significant (p-values > 0.05) levels. Data were ranked by stock composition because this is the value most widely reported in the literature. These analyses were also performed on an individual stock basis for five frequently encountered stocks to reveal perspectives over a variety of stock richness and abundance conditions. For a sub-set of those stocks, contour plot representations were created for comparison to SSCPUE contour plot results. Finally, scatterplots with linear trends were created to visualize and aid interpretation of data. The statistical package Wessa [42] was used for Kendall’s τ analyses.

## Results

### At-sea data collection and sampling

Fisheries data and samples were collected from 38 of the 40 pre-defined month-area strata with only KO/May and KC-n/May lacking data. A total of 2,651 vessel-days effort yielded 9,584 Chinook salmon encounters (Fig 1, Tables 1 and 2). Fishing effort (and samples) were unevenly distributed across space and time. Within month-area strata, the number of vessel-days effort ranged from 7 to 205 (mean = 62, median = 55; Table 1). Greater fishing effort occurred in the north where fisheries were open, and lower in the south where non-retention sampling predominated. In CA, sampling effort trended higher during the open or partially-open time-area strata (FB and southward/July; FB/August). The overall numbers of sampling days conducted using non-retention (1,198, 45%) and retention (1,453, 55%) sampling techniques were similar, but non-retention sampling in CA fisheries represented 73% of days fished (1,079 of 1,477 days) whereas OR fisheries had only 10% of sampling conducted as non-retention (119 of 1,174 days). The number of vessels used for sampling was similar in CA (N = 88) and OR (N = 89).

**Table 1:** Numbers of vessel-days of salmon troll fishing effort for 2010 at-sea sampling across nine spatial strata.

	May		June		July		August		September	Totals	
	non-retention	Retention	non-retention	retention	non-retention	retention	non-retention	retention	non-retention	non-retention	retention
NO		75.29		176.96		73.86		181.35	33.00	33.00	507.45
CO		108.71		166.08		34.14		204.65	30.07	30.07	513.58
KO		0.00	34.96			7.00		27.00	20.93	55.89	34.00
KC-n	0.00		37.00		55.00		60.00		60.00	212.00	0.00
FB^1^	9.00		47.00			91.57		120.00	70.00	126.00	211.57
SF-n^2^	24.00		59.00		32.00	60.20	60.00		59.73	234.73	60.20
SF-s^2^	52.00		60.00		38.00	48.19	58.00		67.27	275.27	48.19
MO-n^2^	35.69		42.11		22.00	60.04	40.00		28.00	167.80	60.04
MO-s^2^	8.31		14.89		11.00	18.00	17.00		12.00	63.20	18.00
totals	129.00	184.00	294.96	343.04	158.00	393.00	235.00	533.00	381.00	1197.96	1453.04

^1^ Open July 1–4, 8–11, 15–29, and all of August.

^2^ Open July 1–4, 8–11.

Monthly numbers of non-retention and retention vessel-days of salmon troll fishing effort. A total of nine spatial strata from Cape Falcon, Oregon (OR) to Santa Barbara, California (CA) were sampled from May—September 2010. Area abbreviations (also see Fig 1): North Oregon Coast (NO), Central Oregon Coast (CO), Oregon Klamath Zone (KO), California Klamath Zone-north (KC-n), Fort Bragg (FB), San Francisco north (SF-n) and south (SF-s), Monterey north (MO-n) and south (MO-s).

**Table 2:** Numbers of sub-legal and legal-sized Chinook salmon encounters.

	May		June		July		Aug		Sept		Totals	
	sub-legal	Legal	sub-legal	legal	sub-legal	legal	sub-legal	legal	sub-legal	legal	sub-legal	legal
NO	-	**404**	-	**1102**	-	**403**	-	^**1**^ **532**	7	23	7	2464
CO	-	**453**	-	**616**	-	**75**	-	**601**	31	64	31	1809
KO	-	**0**	-	44	-	**10**	-	**69**	49	86	49	209
KC-n	0	0	6	64	7	127	88	382	121	247	222	820
FB^2^	6	91	10	159	2	**483**	8	**533**	49	441	75	1707
SF-n^3^	10	37	23	87	9	*395*	22	138	13	37	77	694
SF-s^3^	27	86	78	198	17	*99*	5	114	11	70	138	567
MO-n^3^	6	11	3	17	16	*377*	34	114	42	50	101	569
MO-s^3^	0	2	0	5	0	*13*	0	9	0	16	0	45
Totals	49	1084	120	2292	51	1982	157	2492	323	1034	700	8884

^1^ Eight fish encounters were excluded from CPUE calculations (see text for details).

^2^ Open July 1–4, 8–11, 15–29, and all of August.

^3^ Open July 1–4, 8–11.

Monthly numbers of sub-legal and legal-sized Chinook salmon encounters recorded at-sea during 2010. Area abbreviations (also see Fig 1): North Oregon Coast (NO), Central Oregon Coast (CO), Oregon Klamath Zone (KO), California Klamath Zone-north (KC-n), Fort Bragg (FB), San Francisco north (SF-n) and south (SF-s), Monterey north (MO-n) and south (MO-s). Retention fishery sampling is indicated by bold, mixed retention/non-retention fisheries sampling by italic, and non-retention fisheries by regular text.

From the 9,584 Chinook salmon encounters recorded by participating fishermen (Table 2) biological samples were obtained for 9,554 fish. The number of legal-sized Chinook salmon encounters per month-area stratum ranged from 2 to 1,102 (mean = 207, median = 91; includes 28 fish with missing length data estimated as legal-sized). Among strata, average CPUE ranged from 0.24 to 10.11 fish per vessel-day of fishing effort (Table 3). The sampling goal of 200 legal-sized fish per week-area stratum was rarely achieved due to these low catch rates, non-retention permit constraints, and because sufficient numbers of sampling vessels were not always available. Larger sample sizes were generally obtained in areas NO, CO, and FB which had sizeable fleets and more open fishing days. Eight samples included in the fish encounter and GSI data sets were collected by fishermen participating in a similar project; these samples were excluded from CPUE analysis because compatible effort data were not available. After removal of sub-legal sized fish, fish that failed to yield adequate genetic data and identification, duplicate genotypes, and some sampled fish that were a different species (mostly coho salmon), 8,240 individual assignments for legal-sized fish were available for stock composition estimates (S2 Appendix) and SSCPUE calculations (S3 Appendix).

**Table 3:** Observed mean catch per unit effort (CPUE, vessel-day fishing effort).

Area	May	June	July	August	September
NO	**5.37**	**6.23**	**5.46**	^**1**^ **2.89**	0.70
CO	**4.17**	**3.71**	**2.20**	**2.94**	2.13
KO		1.26	**1.43**	**2.56**	4.11
KC-n		1.68	2.31	6.37	4.12
FB	10.11	3.38	**5.27**	**4.44**	6.30
SF-n	1.54	1.47	***4*.*28***	2.30	0.62
SF-s	1.65	3.30	***1*.*15***	1.97	1.04
MO-n	0.31	0.40	***4*.*60***	2.85	1.79
MO-s	0.24	0.34	***0*.*45***	0.53	1.33

^1^ CPUE calculation exclude 8 samples (see text for details).

Observed mean CPUE calculated by dividing the numbers of legal-sized Chinook salmon encounters (Table 1) with vessel-days of fishing effort (Table 2). Retention fishery sampling is indicated by bold, mixed retention/non-retention fisheries sampling by italic, and non-retention fisheries by regular text. Area abbreviations (also see Fig 1): North Oregon Coast (NO), Central Oregon Coast (CO), Oregon Klamath Zone (KO), California Klamath Zone-north (KC-n), Fort Bragg (FB), San Francisco north (SF-n) and south (SF-s), Monterey north (MO-n) and south (MO-s). Data were collected May–September 2010; no data were collected during May in KO and KC-n.

### Comparison to commercial fishery

Project catch and fishing effort provided good coverage relative to the commercial fishery (Table 4). At-sea catch locations represented 21.4% of the total commercial harvest and vessel-days effort were 20.6% of total commercial fishing effort May–August, 2010. The project CPUEs calculated with inclusion and exclusion of zero-catch vessel-days effort (6.11 and 7.98 fish / day, respectively) bracketed that of the commercial fishery (7.69 fish / day; PFMC data does not account for trips with zero catch). Approximately 24.1% of the total commercial fleet that made landings in CA and OR during 2010 participated in this study.

**Table 4:** Comparison between at-sea study and 2010 commercial fishery data.

Data categories	At-sea study	Commercial Fishery	% At-sea study / Commercial fishery
N landed fish, OR	4,482	26,454	16.9%
N landed fish, CA	4,402	15,088	29.2%
N landed fish, total	8,884	41,542	21.4%
Vessel effort, OR	1055	3428	30.8%
Vessel effort, CA	398	1,975	20.2%
Vessel effort, total	1,453	5,403	26.9%
Vessel-days effort excluding days with zero-catch	1,113	as above	20.6%
N participating vessels, OR^1^	78	370	21.1%
N participating vessels, CA	63	215	29.3%
N participating vessels (retention only)	141	585	24.1%
CPUE (legal-sized fish/vessel-day effort)	6.11	7.69	n/a
CPUE (excluding zero-catch days)	7.98	n/a	n/a

^1^ Includes Astoria.

Comparison between the 2010 Oregon (OR) and California (CA) at-sea study and the commercial Chinook salmon troll fishery: numbers (N) of landed fish, vessel-effort measured as N days fished, N vessels that participated in this study and the commercial fishery, and catch per unit effort (CPUE, vessel-day fishing effort). Commercial fisheries data included landings south of Cape Falcon, OR, from May—August 2010 and excludes State Fall area fisheries.

### Statistical modeling of CPUE

#### Time-area variability in CPUE

**Fig 2:**
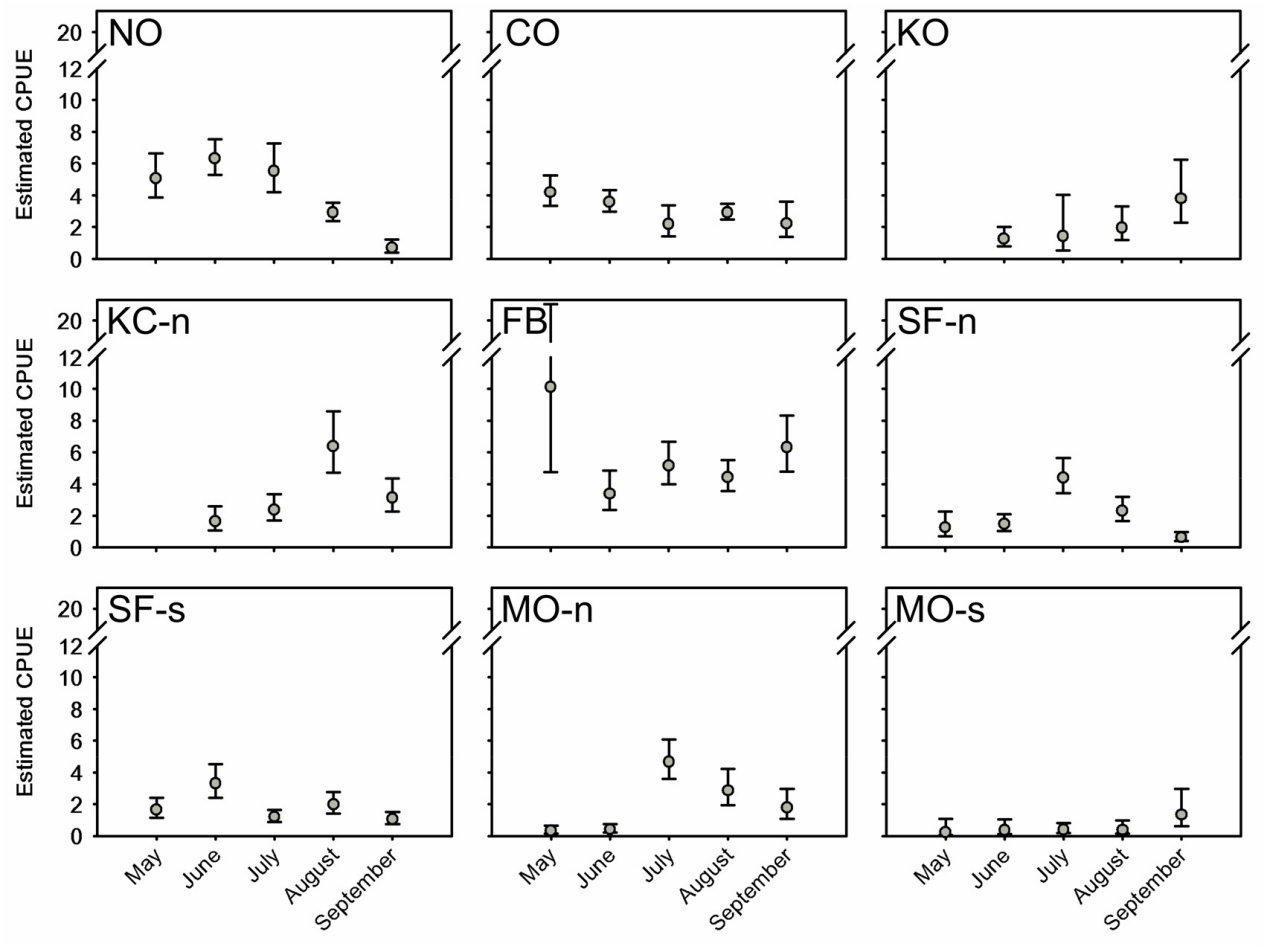
Mean catch per unit effort and 95% confidence intervals. Mean catch per unit effort (CPUE) was modeled for nine area strata using a negative binomial model CPUE ~ month x area. CPUE is the number of legal-sized fish caught per vessel-day fishing effort. Area abbreviations (also see Fig 1): North Oregon Coast (NO), Central Oregon Coast (CO), Oregon Klamath Zone (KO), California Klamath Zone-north (KC-n), Fort Bragg (FB), San Francisco north (SF-n) and south (SF-s), Monterey north (MO-n) and south (MO-s).

The majority of vessel-days effort (n = 2,580 vessel-days, ~97% days fished) met criteria for inclusion in the CPUE modeling data set. The interaction between exogenous variables “time” and “area” explained more variance than either of the model terms considered individually (Table 5). The next strongest term was “area”, followed by “time”. Month-area variability in CPUE, with associated sampling error, is presented in Fig 2 (for week-area results see S1 Fig). In most cases, within-area changes in CPUE followed gradual trends over time. Within the northernmost two strata, mean CPUEs were higher early in the season and then trended downward (NO) or stabilized (CO). In the KO and KC-n, mean CPUE started low early in the season and then increased, with August/KC-n showing a transient peak. The area FB, May, mean CPUE was higher than any other sampled area. After dipping in June, the CPUE showed a zigzag pattern for the remainder of the season. The area MO-s had lowest overall CPUE relative to all other strata. The within-area weekly CPUE results mostly correspond to monthly patterns, but moderate fluctuations and occasional abrupt changes in CPUE are apparent (S1 Fig). Although the week x area model is technically a better fit to the data, the broader month time-scale provides larger, more representative sample sizes (both for fisheries sampling and GSI) and balances the effect of outlier weeks on CPUE results.

**Table 5:** Models of catch per unit effort.

Model	Model Terms	AIC or (ΔAIC)	Residual Deviance	Deviance decrease	Deviance decrease p-value
Null		11842	2760 on 2579 df		
Month x Area		(- 475)	2741 on 2537 df		
	Month			20.3	0.0005
	Area			279	< 2.2 e -16
	Month x Area			333	< 2.2 e -16
Week x Area		(- 637)	2719 on 2415 df		
	Week			128	< 2.2 e -16
	Area			302	< 2.2 e -16
	Week x Area			682	< 2.2 e -16

Log-linear negative binomial models (CPUE ~ time x area) were used to evaluate the explanatory power of terms “time” and “area” and a term for their interaction on estimated mean catch per unit effort (legal-sized fish encounters per vessel-day fishing effort). Model fit was assessed by calculating the Akaike Information Criteria (AIC) score for a null model and evaluating the change in AIC score (deltaAIC, ΔAIC) for a model with terms. An ANOVA model was used to determine if each model term was a significant effect.

#### Effect of non-retention and retention sampling technique on CPUE

**Fig 3:**
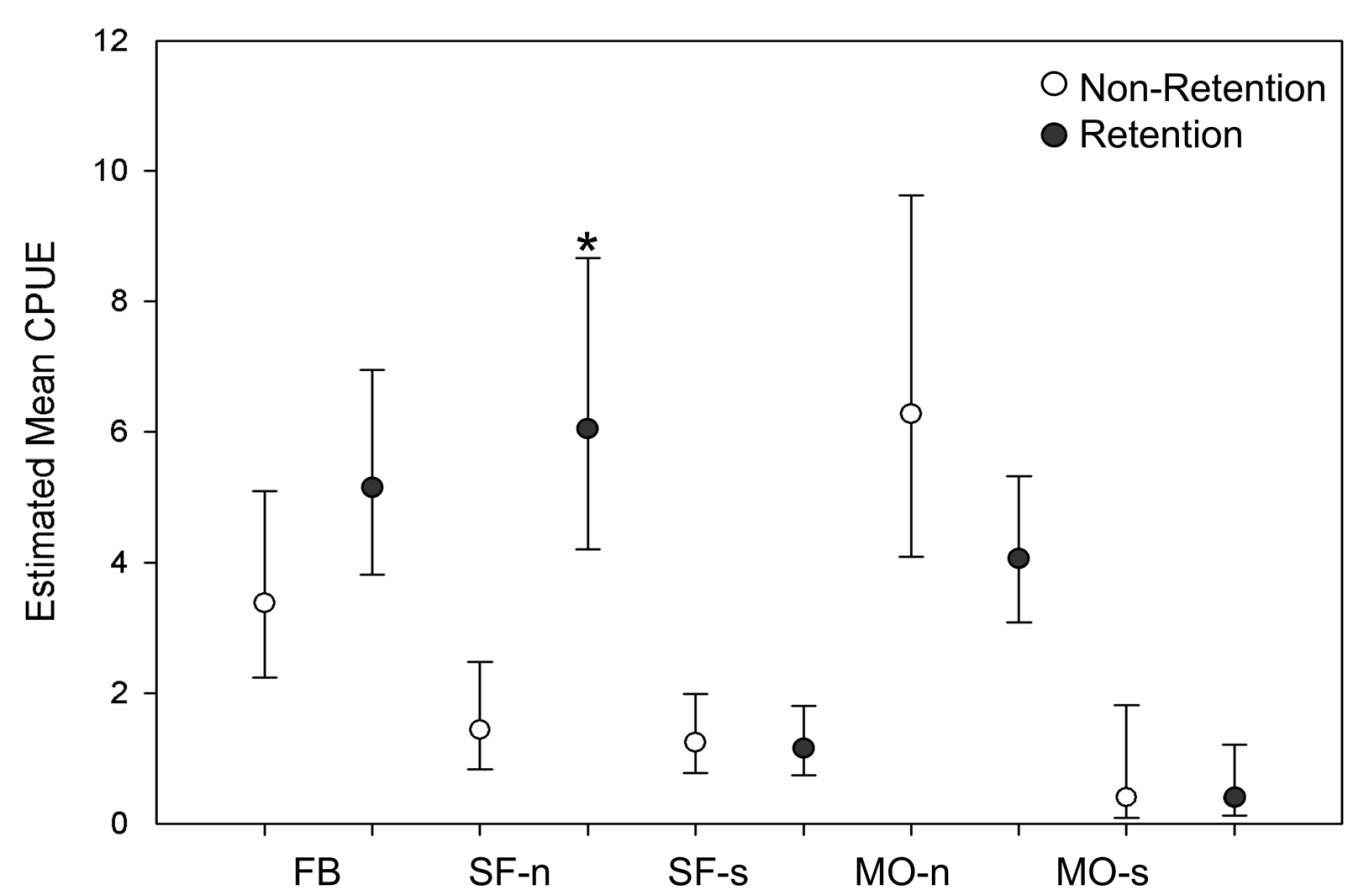
Mean catch per unit effort (CPUE, vessel-day fishing effort) for non-retention and retention fisheries sampling. CPUE was compared within each of five sampling areas: Fort Bragg (FB), San Francisco north (SF-n) and south (SF-S), Monterey north (MO-n) and south (MO-s). For FB, the June/non-retention fishery was compared to the July/retention fishery. For remaining areas, the July 1–4 and 8–11 retention fishery was compared to non-retention sampling conducted over the remainder of July. Mean CPUEs and 95% confidence intervals were calculated using a log-linear negative binomial model. The null hypothesis of no difference between CPUE for retention fishery is rejected at a probability of z > .0.5, denoted by *.

An overall effect of fisheries sampling technique on catch rates was not strongly supported by statistical analyses (Table 6, Fig 3). Only in the SF-n area was estimated mean CPUE significantly higher in the retention than the non-retention fishery. Non-significant trends within the remaining four areas were inconsistent: estimated CPUE was higher in the retention than non-retention fishery in area FB, approximately equal within areas SF-s and MO-s, and lower in area MO-n. We found no support (Chi-square = 0.0098, df = 1, p-value = 0.9211) for a significant difference between sampling techniques as measured by the proportion of successful and unsuccessful fishing days in the July MO-n, MO-s, SF-n, and SF-s fisheries data set. However, a greater proportion of zero-catch days was identified for the FB/June non-retention than for the FB/July retention fishery (Chi-square = 8.68, df = 1, p-value = 0.0032). Given the overall weak support effect of fisheries sampling technique on catch we treat these two types of data as approximately equal in subsequent analyses.

**Table 6:** Comparison of catch per unit effort (CPUE) for non-retention and retention fisheries sampling techniques.

	Estimated Model Coefficients					Negative Binomial Model Results			
Area	Fishery	N days fishing	Mean	Lower CI	Upper CI		Std. Error	z value	p-value
FB	Non-retention	47	3.38	2.24	5.09	(Intercept)	0.208	5.847	5.00 e-09
	Retention	83	5.14	3.81	6.94	Fishery R	0.259	1.622	0.105
SF-n	Non-retention	32	1.44	0.83	2.48	(Intercept)	0.278	1.307	0.191
	Retention	57	6.04	4.21	8.66	Fishery R	0.333	4.306	1.66 e-05
SF-s	Non-retention	38	1.24	0.77	1.99	(Intercept)	0.247	0.862	0.389
	Retention	45	1.15	0.74	1.80	Fishery R	0.338	-0.192	0.848
MO-n	Non-retention	22	6.27	4.09	9.62	(Intercept)	0.218	8.419	< 2 e-16
	Retention	59	4.05	3.08	5.32	Fishery R	0.258	-1.692	0.091
MO-s	Non-retention	10	0.40	0.09	1.82	(Intercept)	0.775	-1.182	0.237
	Retention	18	0.39	0.12	1.21	Fishery R	0.969	-0.029	0.977

Comparison between CPUE (vessel-day fishing effort) for individual areas sampled using non-retention and retention techniques. Difference in CPUE was evaluated using a log-linear negative binomial model, rejecting the null hypothesis of no difference between CPUE at a probability of p < 0.05 (Fishery R; shown in bold). In areas San Francisco (SF-n, -s) and Monterey (MO-n, -s) the July 1–4 and 8–11 retention fishery was compared to the July 12–31 non-retention fishery. For area Fort Bragg (FB), the retention fishery conducted on July days 1–4, 8–11, and 15–29 was compared to the June non-retention fishery.

#### Effect of fisherman on CPUE

The model with fishermen effect as the only independent variable (ΔAIC = -648, residual deviance = 2741 on 2401 degrees of freedom) was a slightly better fit to the data than the week x area model (Table 5). The term fishermen effect was significant on the model (ANOVA, p < 2.2e-16) but, in the majority of cases (n = 131 of 172), the estimated CPUE among individual fishermen was not significantly different. For the 41 fishermen with statistically different CPUE, it was higher for 19 (uncorrected p-values from 0.0004 to 0.0488) and lower for 22 (uncorrected p-values 0.0000 to 0.0400), relative to the arbitrarily set reference required by the model. The modest differences among fisherman CPUE suggests that differences among fisherman ability has limited consequences for CPUE-based abundance estimates at the scale measured here.

### Comparison between genetic assignments to CWT recoveries

Genetic stock assignments were mostly concordant with stock of origin as identified by recovery of CWTs from fish in OR (51 total, S4 Appendix). Correct genetic assignment to region of origin was made for 35 of the 38 fish (92%) that met the posterior probability criteria (≥ 90%). Seven reporting regions were represented in the 38 fish sample, with 100% correct allocation to five (CA Central Valley fall, Lower Columbia fall, Mid Columbia Tule, Rogue, and Upper Columbia summer/fall stocks) of these seven regions. The Snake River fall hatchery stock was represented by seven fish, five of which correctly assigned and two of which mis-assigned to Deschutes fall and N. Puget Sound reporting units. The third mis-assigned fish was a N. OR Coast fish that allocated to the Mid OR Coast reporting units. No tagged fish were available for comparison in CA, but the concordance rate between assignments with the SNP baseline and CWT recoveries was 98.95% for over 1,000 fish port sampled from CA fisheries in 2010 [30].

### Stock richness, distribution and CPUE-based abundance patterns

**Fig 4:**
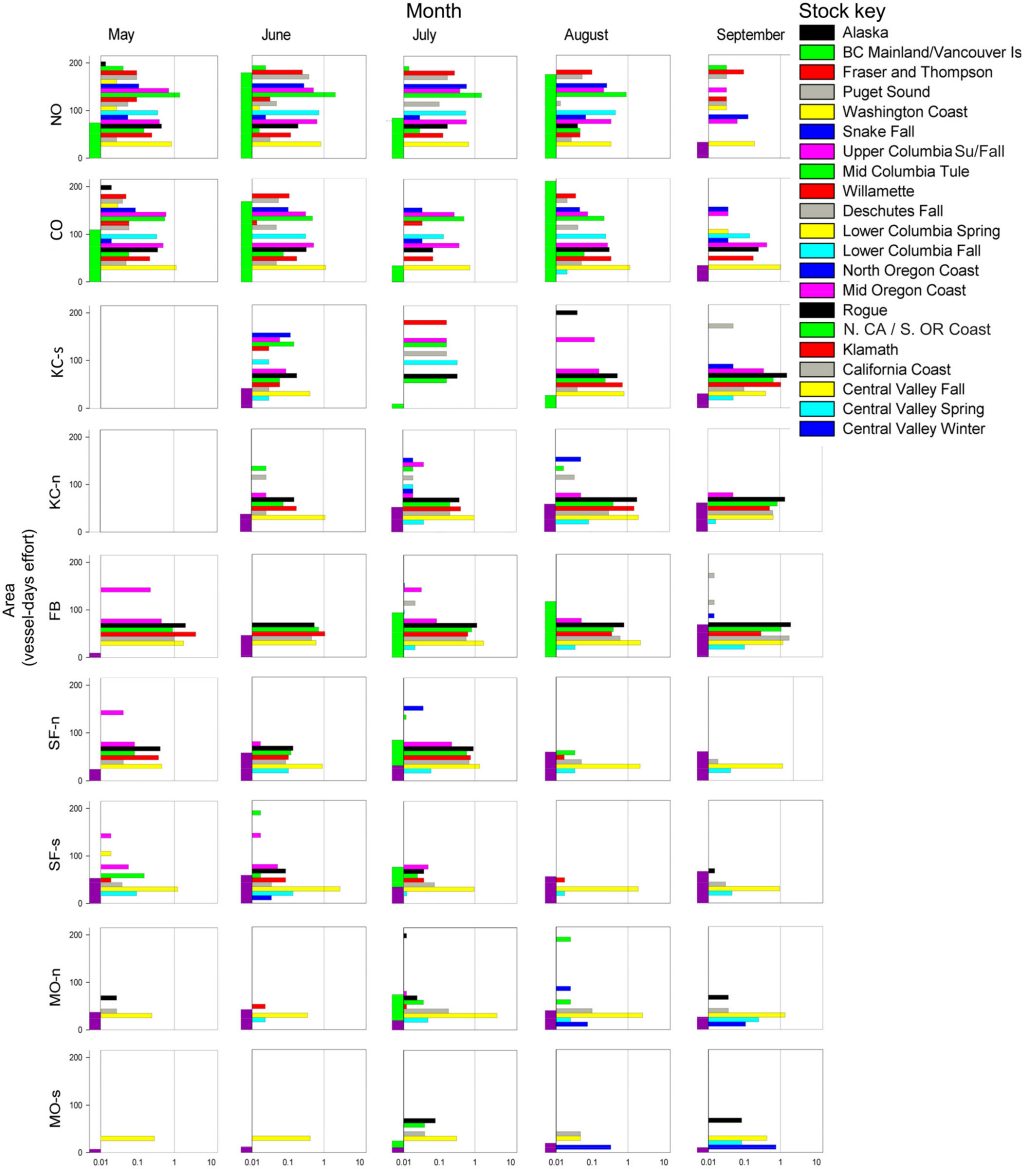
Log stock-specific catch per vessel day of fishing effort. Stock-specific catch per unit effort was sampled for 22 stocks encountered in nine area strata sampled May—September, 2010. Vertical green (retention) and magenta (non-retention) bars on left axis shows effort in total days fished. Stocks are listed by north to south order of natal rivers. Sample area abbreviations (also see Fig 1): North Oregon Coast, NO; Central Oregon Coast, CO; Oregon Klamath Zone, KO; California Klamath Zone north, KC-n; Fort Bragg, FB; San Francisco north, SF-n; San Francisco south, SF-s; Monterey Bay north MO-n; and Monterey Bay south, MO-s. Sampling was not conducted during the month of May in areas KO and KC-n.

**Fig 5:**
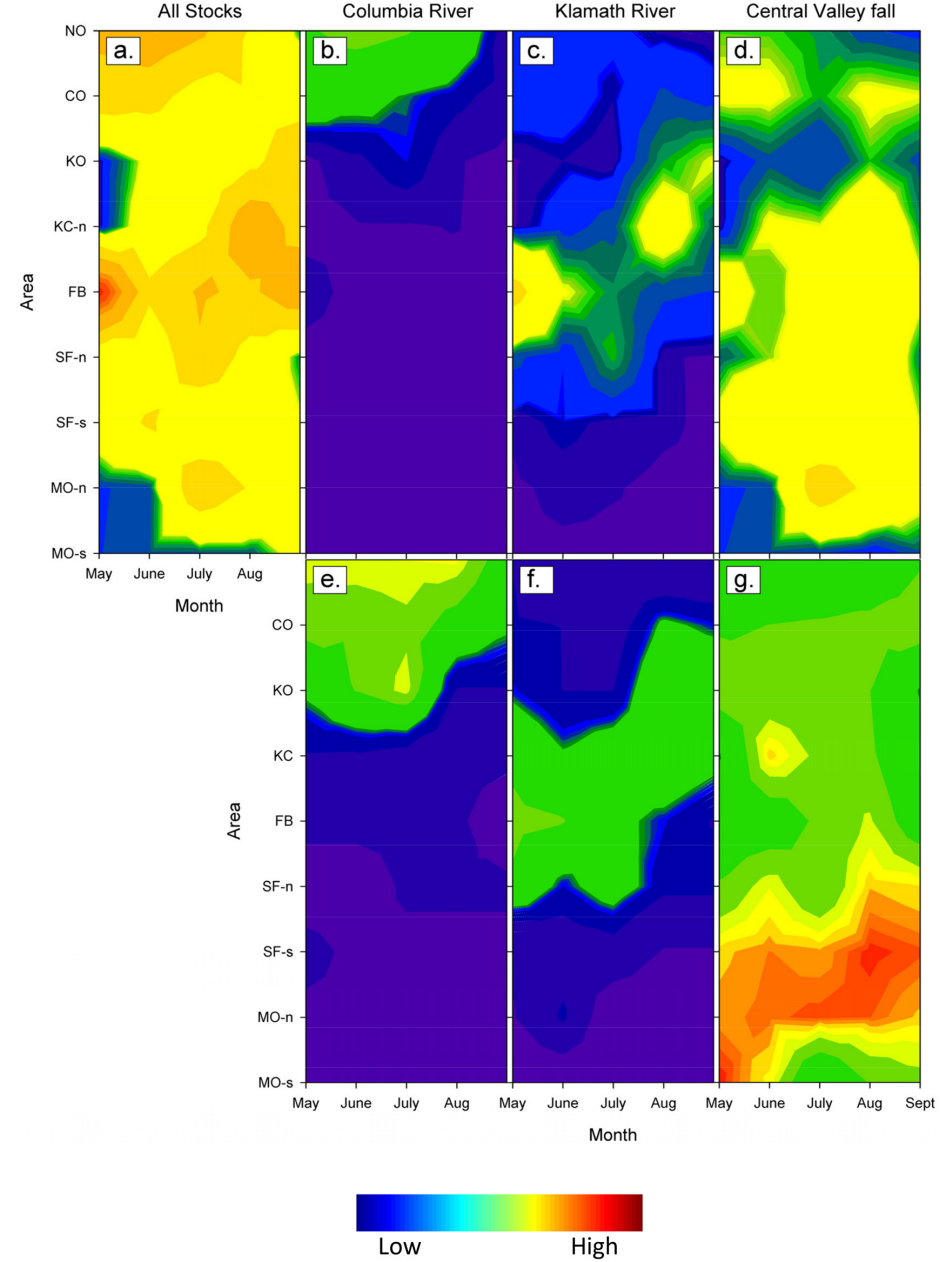
Contour plots of Chinook salmon log-catch per unit effort and genetic stock composition. Log catch per vessel-day fishing effort (CPUE) (a–d) and stock composition estimates (e–h) are presented for nine area (y-axis) and five month (x-axis) strata. Results are shown for all stocks (CPUE only), and for stock groupings Columbia River/Snake complex, Klamath, and California Central Valley fall. See Fig 1 and text for area abbreviations and sampling details. No sampling was conducted in KO/May and KC-n/May and KC-s, all season.

Stock richness was highest in areas sampled to the north and trended downward to the south (Fig 4). Nearly 1/3 of the 22 stock groups encountered (n = 7) originated from the Columbia River (Columbia/Snake stock complex). Those stocks were distributed primarily to the north, and had SSCPUEs that decreased towards the end of the sampling season. The CA Central Valley fall stock was widely distributed, but showed transient peaks of abundance in areas FB and KC-n. This was the only stock present across nearly all sampled strata, and its SSCPUE values were approximately equal to or greater than most of the other stocks. Stocks originating from near the OR-CA border (e.g., Rogue, Klamath and CA Coastal) tended to have higher SSCPUEs in areas proximal to their natal river mouths (KC-n, FB, SF-n). The CA Central Valley winter stock was unique in that it was detected only in southern sampling areas, showing a slight increase in SSCPUE during the month of September. Spatial and temporal patterns in SSCPUE can be inferred from Fig 4, but results are more easily interpreted when visualized as log-CPUE contour plots (Fig 5). Peaks in CPUE are shown as warm colors (“hot spots”), while areas with low CPUE use cooler colors (green to blue). Comparisons between the all-stock and individual stock panels reveal which stocks contributed to areas of high CPUE. The contour plot patterns for SSCPUE and stock composition correspond fairly well for stocks with limited distributions, but correspondence is reduced for stocks (e.g., CA Central Valley fall) that are broadly distributed. The contours between KO and KC-n /May does not reflect stock distribution, as the contour plot smoothing algorithm fills in missing data.

### Correlations between stock composition data and SSCPUE

**Fig 6:**
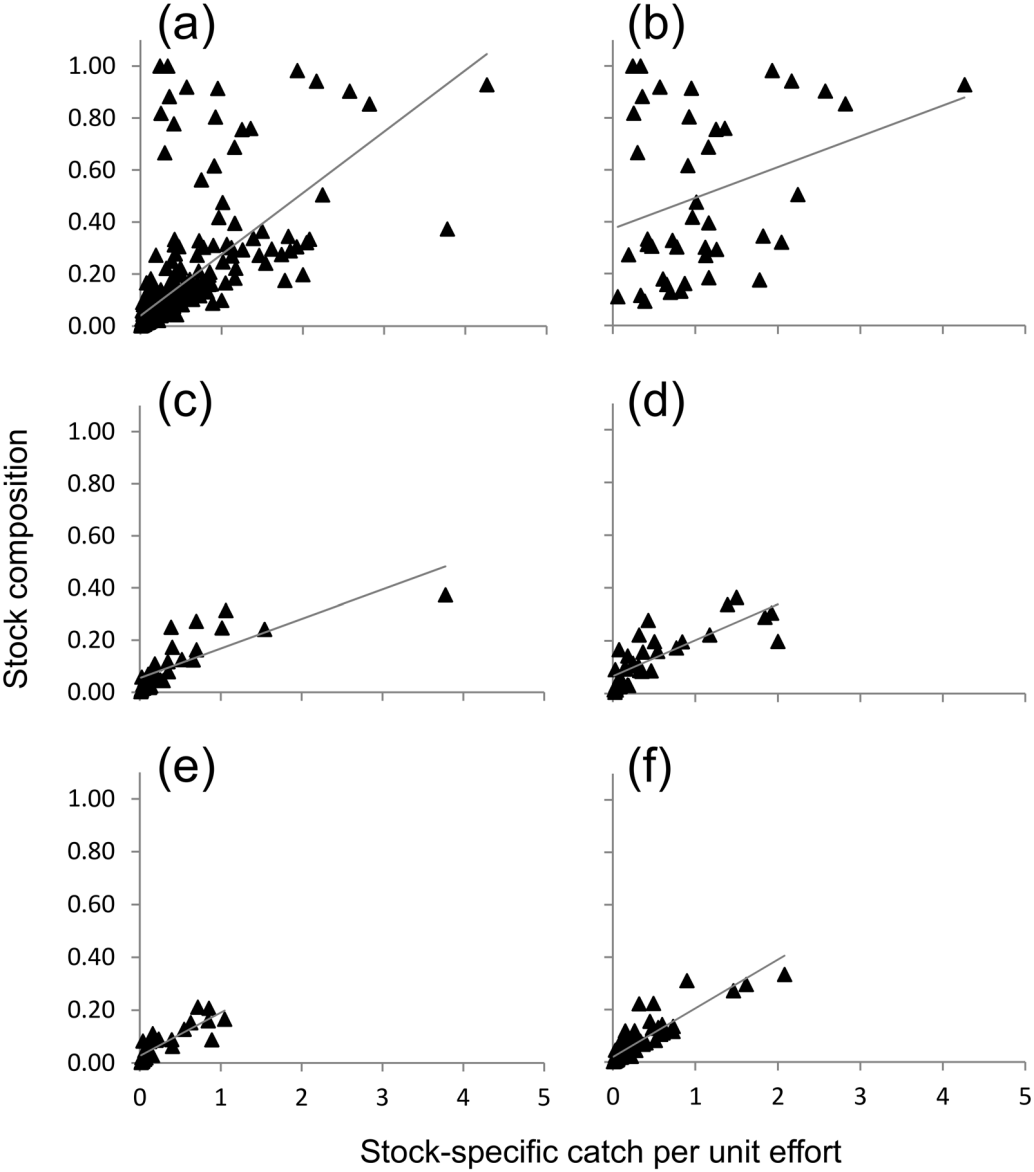
Scatterplots of paired stock-specific catch per unit effort (SSCPUE) and stock composition measures. Data are presented on a month-area basis for all stocks (a) and individual stocks (b) California Central Valley fall, (c) Klamath, (d) Rogue, (e) California Coastal, and (f) Northern California/Southern Oregon Coastal.

The measures of stock composition and SSCPUE for the all-stock data set were significantly correlated when evaluated over the full range of values but, at fairly low threshold points (≥ 17.32% stock composition), Kendall’s τ values decreased and p-values increased to non-significant levels (Table 7). Scatterplots for all stocks (Fig 6a) and five individual stocks—CA Central Valley fall, Rogue, Klamath, CA Coastal, and Columbia/Snake River complex (Fig 6b–6f)—show that spread between data points becomes greater as each measure increased. For the latter four stocks, the spread between points is more prevalent across the SSCPUE than stock composition axis. The dominant stock in the all-stock data set, CA Central Valley fall (identified by comparing Fig 6a and 6b), was distinguished by widely fluctuating stock composition and SSPUE measures. This was the only stock for which Kendall’s τ correlation analysis failed to show support for an association between SSCPUE and stock composition over the entire range of values. Stock composition estimates for this stock ranged from < 15% (e.g., in NO/May and June; KO/July, September) to > 90% in some of the southern mixed stock fishery samples (e.g., SF-s/August, September; MO-n/July; S2 Appendix) despite relatively low SSCPUE values in those regions (Fig 4, S3 Appendix). The inclusion of samples collected from areas characterized by wide differences in stock richness was a driving factor in the discord between stock composition and SSCPUE values.

**Table 7:** Correlation analyses for paired genetic stock composition and stock-specific catch per unit effort (SSCPUE) values.

	Full data set		Threshold data set		
	τ	p-value	τ	p-value	min. % stock composition
All stocks	0.739	0.000	0.149	0.06	17.32
California Central Valley fall	0.189	0.081	n/a	n/a	0.00
Rogue	0.659	<0.001	0.385	0.076	16.03
Klamath	0.721	0.000	0.360	0.178	12.47
CA Coastal	0.568	<0.001	0.500	0.108	9.89
Columbia/Snake complex	0.752	0.000	0.454	0.062	12.91

Strengths of associations, evaluated using Kendall’s τ correlation coefficients, between paired genetic stock composition and stock-specific catch per unit effort (SSCPUE, vessel-day effort) values. Data were collected from nine month-area strata sampled May–September, 2010. The full data set includes all non-zero pairs of data, in contrast to the threshold data set which includes only pairs of data above a minimum stock composition value (min % stock composition). The threshold data set represents the stock composition threshold point above which that value failed to correlate (p-values > 0.05) with SSCPUE. Analyses were performed using data from all stocks and on an individual-stock basis for five stocks (or stock groupings) that represent a range of sample collection conditions.

## Discussion

In this study, we provide the first comprehensive assessment of fine-scale, geo-referenced ocean distribution patterns among genetically distinct Chinook salmon stocks as they migrate within the southern California Current large marine ecosystem. Such a perspective enables unique insights into dynamic spatial and temporal shifts of relative abundance, as indexed by CPUE, of multiple stocks at a scale that encompasses most of the range of typical migratory patterns for south-migrating Chinook salmon stocks. The individual-based stock-, region- and time-specific approach presented here makes significantly more dense and focused information available for the study of Chinook salmon migration behavior than previously possible using physical tags (e.g., CWTs) or genetic stock composition data alone [43–45].

The SSCPUE and stock composition estimates are two complementary measures for tracking fish distribution. Stock composition characterizes the relative proportion of stocks present in a single sample, while SSCPUE provides a measure of abundance for each of the stocks across fisheries. While stock composition values are likely to be poor representatives of relative stock abundance estimates, scientific literature has not previously described or statistically evaluated the degree of discord between these two measures using empirical data. Using the non-parametric Kendall’s τ, the two indices were significantly correlated when all stocks were considered, but the correlation disappeared when locally rare stocks were excluded. Rare stocks inflate τ values because their stock composition and SSCPUE measures will always be ranked low relative to the full range of available values. Moreover, rare stocks exert little influence on other stocks’ composition estimates and have no effect on SSCPUE. Discord between SSCPUE and stock composition indices was strongly influenced by comparison among samples collected from areas with wide differences in stock richness. Results from the CA Central Valley fall stock best exemplify this point: in southern areas, the composition values were disproportionately high, despite low abundance, simply because fewer other stocks were present than in the north. While it may be intuitive to equate high stock composition values with high abundance, that was clearly not the case for the CA Central Valley fall stock. Because SSCPUE is unbiased by other stocks present in fishery samples, it is more easily interpreted across multiple time-area strata. Thus, SSCPUE is a superior measure for tracking movements of individual stocks or comparing local abundance across time-area strata.

Some of the patterns of stock distribution we show are similar to trends known from CWT data [44], but SSCPUE data allows us to identify a greater number of stocks [9,30,46] at much higher spatial and temporal resolution than typical CWT dock-side sampling programs. Our results show a clear increase in stock richness from south to north, a trend that persisted even with low sample sizes for some time-area strata (e.g., KO/July). The greater number of stocks observed in northern sampling areas reflects overlap in distributions of stocks that breed to the north (e.g., Columbia River, Puget Sound, and some Canadian stocks) and to the south (e.g., California Central Valley fall, Klamath, and Rogue) of the sampled area (Fig 4). The contour plot results provide a simplified picture of stock distributions, and aid contrasting SSCPUE and stock composition indices. For example, the Central Valley fall SSCPUE (Fig 5d) and stock composition (Fig 5g) contour plots show distinctly different patterns. Visualizations such as these could help fishery managers and fishermen develop directed fishing strategies. An individual stock “hot spot”, such as the one observed for the FB/May stratum, could be targeted by fishermen if the predominant stock is one that can withstand fishing pressure. Or, perhaps fishing could be shifted away from a given area if the hot spot represents an aggregation of fish for which conservation is a concern. A web portal (FishTrax, fp.pacificfishtrax.org/portal) was created for this study to generate customizable, stock-specific catch and effort distribution maps, with data being continually updated during the season. This portal is available to fishermen, managers, and the general public.

Commercial fishery restrictions necessitated the use of non-retention sampling for most of the season in California, and all areas in September. Determining whether the CPUE of retention and non-retention sampling differs is important because CPUE-based estimates of fish density assume that fish contact rates are proportional to abundance. Fluctuations in catch can, however, occur from differences in fleet efficiency, the environment, and dynamics of the fish population [47]. We predicted that CPUE of retention fisheries would be higher than non-retention fishery because incentives to catch fish are greater for fishermen who are able to retain and sell their catch (non-retention samplers were compensated a fixed rate per day). Additionally, fishermen cooperate amongst themselves by sharing location and catch information; that type of information is reduced in non-retention fisheries because fewer fishermen are on the water searching for fish. Our analyses show that CPUE and stock compositions from non-retention fisheries were consistent with similar retention fisheries. The non-retention sampling enabled continuity of fishery catch data while reducing mortality rates on sampled fish. Only lack of sampling in KO and KC in May created discontinuities in the data, but even small gaps such as those can render interpretation of contour plot data more difficult. Overall, we successfully characterized stock distribution across a vast swath of ocean and demonstrate that non-lethal sampling in the ocean is feasible for assessing distributions and abundance.

Current fishery models for stock assessment and harvest management are built around a CWT sampling program and monitoring of adults returning to freshwater. The data provided by GSI techniques differ substantially from those provided by the CWT program, including in statistical properties (sources of error and uncertainty), ability to determine brood year (age), and representation of stock aggregations. Statistical uncertainty in salmon models arises from expansion of tag recoveries, mark rates, and incorporation of sampling and fishery effort. For CWT-based models, expansions are based on few observations and low mark rates, leading to high uncertainty in the CWT-based estimates of the stocks they are intended to represent. GSI estimates, in contrast, provide stock-origin data for nearly every sampled fish, eliminating the need for a mark-rate expansion factor. That, coupled with at-sea sampling which yields precisely known effort, further reduces statistical uncertainty. However, for GSI, the accuracy of stock-origin is substantially different from the CWT system. For CWT-marked fish, stock-origin accuracy is near-100% and age-cohort information is provided. In contrast, correct assignment of individuals to populations by GSI depends on the genetic baseline’s ability to discriminate among stocks (e.g., [38,48]), and a 90% correct assignment threshold [7,8] is commonly used for delineation of baseline populations. Genetic baseline power is routinely assessed through 100% mixture simulations, leave-one-out tests of proportional allocations, and empirical tests of GSI-CWT concordance [8,30,38]. Our GSI-CWT concordance test is illustrative of variations among stock-assignment accuracy: the majority of fish were correctly allocated to their reporting groups, but the Snake River fall stock, in particular, mis-allocated to other reporting units, consistent with findings of previous power analyses [40]. We were able to achieve a moderate level of sampling relative to the commercial fishery (e.g., > 20%), similar to the target sampling rate for CWTs (of which only a small proportion actually contain tags). GSI-based sampling programs would need to be designed to collect randomized samples, or at least to distribute sampling over space and time, for the data to be used in the same way as those derived from the CWT program.

Most fisheries models rely on age-specific cohort reconstruction data obtained from CWTs, but GSI does not provide age information directly. Aging can be achieved concurrently through analysis of the scales of salmon that are also genetically identified, although this is slow, expensive, and can be difficult for maturing fish, as they start to reabsorb their scales. This obstacle can be overcome through the use of pedigree-based genetic methodologies (i.e. intergenerational, or parentage-based, tagging) that yield cohort and stock data similar to those obtained through the CWT program [49,50]. Both CWTs and genetic tagging require access to a large and known proportion of the juvenile cohort or spawners in a stock, respectively, so are generally only applied to hatchery stocks. However, GSI and genetic tagging can use the same genotype data: in that case, GSI can be used to identify natural stocks as well as hatchery stocks, and the need to assign “indicators” for predominantly natural stocks is eliminated. Using data that overlapped with those from this study, Satterthwaite and colleagues [51] inferred ocean distribution from spatial variation in CPUE to evaluate the performance of the data-rich Klamath fall stock as a proxy for the data-poor (unmarked) California Coastal stock. The two stocks had similar distributions early in the fishing season, but diverged late in the summer. There are no CWTs used in the California Coastal Chinook stock, so this analysis was only possible with GSI data of the type described here.

## Conclusions and Future Applications

Coordinated, geo-referenced sampling on a large spatial and temporal scale enabled high-resolution assessment of stock-specific abundance and distribution of migrating Chinook salmon in the California Current marine ecosystem. Stock richness was highest in the northern sampling areas and declined to the south. A limited number of stocks were encountered in the southern limits of Chinook salmon’s ocean range. Comparison of stock composition and SSCPUE estimates indicate these measures diverge for stocks present at moderate abundance levels in a fishery. Using effort-adjusted abundance estimates, such as SSCPUE, for quantification of stock distribution yields information that is comparable across fishery samples. In contrast, stock composition results inconsistently corresponded to abundance measures and are not comparable across fishery samples. We show that CPUE of retention and non-retention fisheries was similar and non-retention sampling therefore holds potential for unbiased tests of stock abundance. Conducting test fisheries for pre- or in-season assessments of SSCPUE could lead to strategies that allow maximum sustainable harvest while achieving conservation objectives. While GSI data are not without limitations, the incorporation of CPUE-based stock abundance into fisheries management (and other disciplines) remains a promising and exciting field of opportunity. This study provides proof of concept for implementing at-sea GSI sampling into a coast-wide program for fisheries applications.

## Supporting Information

S1 Fig Catch per unit effort (CPUE, vessel-day fishing effort) estimated on a weekly basis for nine sampling areas. The CPUE and 95% confidence intervals were estimated using a log-linear negative binomial model with terms “week”, “area”, and a term for their interaction. Data were collected May–September 2010. Area abbreviations are: North Oregon Coast (NO), Central Oregon Coast (CO), Oregon Klamath Zone (KO), California Klamath Zone-north (KC-n), Fort Bragg (FB), San Francisco north (SF-n) and south (SF-s), Monterey north (MO-n) and south (MO-s). See text and Fig 2 for details on sample sizes and data collection.
(TIFF)

S1 Appendix List of regions and populations in the Genetic Analysis of Pacific Salmonids (GAPS) baseline v3. Data includes run time, hatchery (H) or wild (W) origin, life stage, collection data, and analysis laboratory (regional allocations based on Seeb and colleagues [8]).
(DOC)

S2 Appendix Genetic stock composition results. Nine area strata sampled consecutively for five months from May–September 2010. Samples from 8,240 legal-sized Chinook salmon were genotyped and matched to standardized microsatellite (n = 3,866, Oregon) or single-nucleotide polymorphism baseline (n = 4,374, California).
(DOC)

S3 Appendix Stock-specific catch per unit effort (vessel-day fishing effort) results for Chinook salmon sampled off the coasts of Oregon and California during 2010. Fish were sampled during open commercial fisheries or from closed areas using non-retention sampling techniques.
(DOC)

S4 Appendix Comparison between genetic stock identification and coded-wire-tag (CWT) stock-origin data for hatchery Chinook salmon marked with a CWT and recovered in Oregon’s 2010 commercial salmon fishery. (DOC)
