# Supplementary figures and images for: Geo-Referenced, Abundance Calibrated Ocean Distribution of Chinook Salmon (Oncorhynchus tshawytscha) Stocks across the West Coast of North America
Source: PLoS One. 2015 Jul 22;10(7):e0131276. doi: 10.1371/journal.pone.0131276 (PMC4511799; doi:10.1371/journal.pone.0131276)

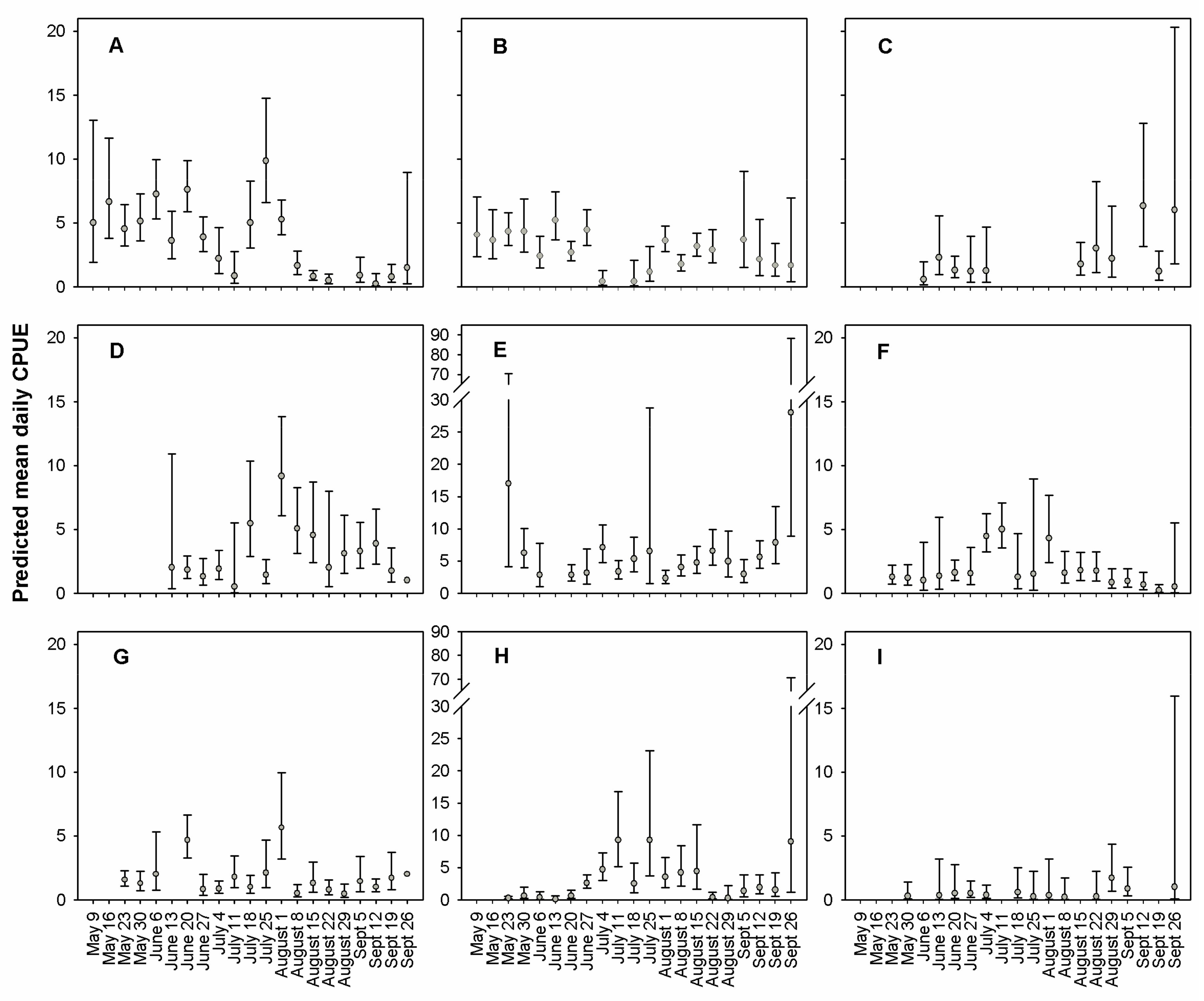

Supplement: S1 Fig — The CPUE and 95% confidence intervals were estimated using a log-linear negative binomial model with terms “week”, “area”, and a term for their interaction. Data were collected May–September 2010. Area abbreviations are: North Oregon Coast (NO), Central Oregon Coast (CO), Oregon Klamath Zone (KO), California Klamath Zone-north (KC-n), Fort Bragg (FB), San Francisco north (SF-n) and south (SF-s), Monterey north (MO-n) and south (MO-s). See text and Fig 2 for details on sample sizes and data collection. (TIFF) [file pone.0131276.s001.tiff]
