# Supplementary material for: Geo-Referenced, Abundance Calibrated Ocean Distribution of Chinook Salmon (Oncorhynchus tshawytscha) Stocks across the West Coast of North America
Source: PLoS One. 2015 Jul 22;10(7):e0131276. doi: 10.1371/journal.pone.0131276 (PMC4511799; doi:10.1371/journal.pone.0131276)
Supplement: S1 Appendix — Data includes run time, hatchery (H) or wild (W) origin, life stage, collection data, and analysis laboratory (regional allocations based on Seeb and colleagues [8]). (DOC) [file pone.0131276.s002.doc]

Appendix 1. List of regions and populations in GAPS (Genetic Analysis of Pacific Salmonids) baseline v 3. Run time, H. (H) or wild (W) origin, life stage, collection data, and analysis laboratory are given (from Seeb et al. 2007 and unpublished data).

| Region  # |  | Population | Run time^1^ | Origin | Life Stage | Collection Date | Analysis  Laboratory^2^ |
| --- | --- | --- | --- | --- | --- | --- | --- |
| 1 | Central Valley fall | Battle Creek | Fa | W | Adult | 2002, 2003 | SWFSC |
|  |  | Feather H. fall | Fa | H | Adult | 2003 | SWFSC |
|  |  | Stanislaus River | Fa | W | Adult | 2002 | SWFSC |
|  |  | Tuolumne River | Fa | W | Adult | 2002 | SWFSC |
| 2 | Central Valley spring | Butte Creek | Sp | W | Adult | 2002, 2003 | SWFSC |
|  |  | Deer Creek spring | Sp | W | Adult | 2002 | SWFSC |
|  |  | Feather H. spring | Sp | H | Adult | 2003 | SWFSC |
|  |  | Mill Creek spring | Sp | W | Adult | 2002, 2003 | SWFSC |
| 3 | Central Valley winter | Sacramento River winter | Wi | W/H | Adult | 1992 - 1995, 1997, 1998, 2001, 2003, 2004 | SWFSC |
| 4 | California Coast | Eel River | Fa | W | Adult | 2000, 2001 | SWFSC |
|  |  | Russian River | Fa | W | Juvenile | 2001 | SWFSC |
| 5 | Klamath River | Klamath River fall | Fa | W | Adult | 2004 | SWFSC |
|  |  | Trinity H. fall | Fa | H | Adult | 1992 | SWFSC |
|  |  | Trinity H. spring | Sp | H | Adult | 1992 | SWFSC |
| 6 | N California/S Oregon Coast | Chetco | Fa | W | Adult | 2004 | OSU |
| 7 | Rogue River | Applegate | Fa | W | Adult | 2004 | OSU |
|  |  | Cole Rivers H. | Sp | H | Adult | 2004 | OSU |
| 8 | Mid Oregon Coast | Coquille | Fa | W | Adult | 2000 | OSU |
|  |  | Siuslaw | Fa | W | Adult | 2001 | OSU |
|  |  | North Umpqua | Sp | W | Adult | 2004 | OSU |
|  |  | Coos^3^ | Fa | H/W | Adult | 2000, 2005 | OSU |
|  |  | Millicoma^3^ | Fa | H/W | Adult | 2000, 2005 | OSU |
|  |  | Sixes^3^ | Fa | W | Adult | 2005 | OSU |
|  |  | Elk^3^ | Fa | H | Adult | 2004 | OSU |
|  |  | South Umpqua^3^ | Fa | H/W | Adult | 2002 | OSU |
| 9 | North Oregon Coast | Alsea | Fa | W | Adult | 2004 | OSU |
|  |  | Nehalem | Fa | W | Adult | 2000, 2002 | OSU |
|  |  | Siletz | Fa | W | Adult | 2000 | OSU |
|  |  | Salmon^3^ | Fa | W | Adult | 2003 | OSU |
|  |  | Yaquina^3^ | Fa | W | Adult | 2005 | OSU |
|  |  | Necanicum^3^ | Fa | W | Adult | 2005 | OSU |
|  |  | Trask^3^ | Fa | W | Adult | 2005 | OSU |
|  |  | Wilson^3^ | Fa | W | Adult | 2005 | OSU |
|  |  | Kilchis^3^ | Fa | W | Adult | 2005 | OSU |
| 10 | Lower Columbia R. spring | Cowlitz H. spring | Sp | H |  | 2004 | CRITFC |
|  |  | Kalama H. spring | Sp | H |  | 2004 | CRITFC |
|  |  | Lewis H. spring | Sp | H |  | 2004 | CRITFC |
| 11 | Lower Columbia R. fall | Cowlitz H. fall | Fa | H |  | 2004 | CRITFC |
|  |  | Elochoman River | Fa | W | Adult | 1995 | WDFW |
|  |  | Green River | Fa | W | Adult | 2000 | WDFW |
|  |  | Lewis fall | Fa | W | Adult | 2003 | WDFW |
|  |  | Lewis North Fork Su^3^ | Fa | W | Adult | 2004 | WDFW |
|  |  | Sandy | Fa | W | Adult | 2002, 2004 | OSU |
|  |  | Washougal River | Fa | W | Adult | 2005 | WDFW |
| 12 | Willamette River | McKenzie | Sp | H | Adult | 2002, 2004 | OSU |
|  |  | North Santiam | Sp | H | Adult | 2002, 2004 | OSU |
| 13 | Mid Columbia R. tule fall | Spring Creek | Fa | H |  | 2001, 2002 | CRITFC |
| 14 | Mid and Upper Columbia R. spring | American River | Sp | W | Adult | 2003 | WDFW |
|  |  | Carson H. | Sp | H |  | 2001, 2004 | CRITFC |
|  |  | Carson NFH | Sp/Su | H | Juvenile | 2006 | CRITFC |
|  |  | Entiat NFH | Sp | H | Juvenile | 2002 | CRITFC |
|  |  | Granite Creek | Sp | W | Adult | 2005 | CRITFC |
|  |  | Granite Creek | Sp | W | Adult | 2006 | NWFSC |
|  |  | John Day | Sp | W | Juvenile | 2000 | OSU |
|  |  |  |  |  | Adult | 2004 | OSU |
|  |  | Klickitat H. | Sp | H | Adult | 2002, 2006 | CRITFC |
|  |  | Klickitat River | Sp | W | Adult | 2005 | CRITFC |
|  |  | Little Naches | Sp | W | Adult | 2004 | WDFW |
|  |  | Little White Salmon | Sp/Su | H | Juvenile | 2005 | CRITFC |
|  |  | Methow River | Sp | H | Juvenile | 1998, 2000 | CRITFC |
|  |  | Middle Fork John Day | Sp | W | Adult | 2004 | OSU |
|  |  |  | Sp | W | Adult | 2005 | CRITFC |
|  |  |  | Sp | W | Adult | 2006 | NWFSC |
|  |  | Naches River | Sp | W | Adult | 1989, 1993 | WDFW |
|  |  | North Fork John Day | Sp | W | Adult | 2004 | OSU |
|  |  |  | Sp | W | Adult | 2005 | CRITFC |
|  |  |  | Sp | W | Adult | 2006 | NWFSC |
|  |  | Shitike Creek | Sp | H | Juvenile | 2003, 2004 | CRITFC |
|  |  | Twisp River | Sp | W | Adult | 2001, 2005 | WDFW |
|  |  | Upper John Day | Sp | W | Adult | 2004 | OSU |
|  |  |  | Sp | W | Adult | 2005 | CRITFC |
|  |  |  | Sp | W | Adult | 2006 | NWFSC |
|  |  | Upper Yakima | Sp | H | Adult, Mixed | 1992, 1997 1998 | WDFW |
|  |  | Warm Springs H. | Sp | H |  | 2002, 2003 | CRITFC |
|  |  | Wenatchee spring | Sp | W | Adult | 1993, 1998, 2000 | WDFW |
| 15 | Deschutes River fall | Lower Deschutes R. | Fa | W |  | 1999, 2001, 2002 | CRITFC |
|  |  | Upper Deschutes R.^3^ | Su/Fa | W | Juvenile |  |  |
| 16 | Upper Columbia R. summer/fall | Hanford Reach CR | Su/Fa | W |  | 1999 - 2001 | CRITFC |
|  |  | Klickitat River | Su | W | Adult | 1994 | WDFW |
|  |  | Klickitat River | Su/Fa | W | Adult | 2005 | CRITFC |
|  |  | Little White Salmon NFH | Fa | H | Juvenile | 2006 | CRITFC |
|  |  | Lower Yakima River | Su/Fa | W | Adult | 1990, 1993, 1998 | WDFW |
|  |  | Marion Drain | U/Fa | W | Adult | 1998, 1992 | WDFW |
|  |  | Methow R. summer | Su | W |  | 1992 - 1994 | CRITFC |
|  |  | Priest Rapids H. | Su/Fa | H | Adult | 1998 | WDFW |
|  |  | Priest Rapids H. | Fa | H | Juvenile | 1998 - 2001 | CRITFC |
|  |  | Umatilla H. | Su/Fa | H | Adult | 2003 | WDFW |
|  |  |  | Fa | H | Adult | 2006 | CRITFC |
|  |  | Wells Dam | Su/Fa | H |  | 1993 | CRITFC |
|  |  | Wenatchee^3^ | Su | W | Adult | 1993 | WDFW |
| 17 | Snake River fall | Clearwater River | Fa | W | Adult | 2000 - 2002 | CRITFC |
|  |  | Lyons Ferry | Fa | W | Adult | 2002, 2003 | WDFW |
|  |  | Nez Perce Tribal H. | Fa | H | Adult | 2003, 2004 | CRITFC |
| 18 | Snake River spring/summer | Bear Valley | Sp | W | Juvenile | 2006 | IDFG |
|  |  | Big Creek | Sp/Su | W | Adult | 2001 - 2003 | CRITFC |
|  |  | Camas Creek | Sp | W | Juvenile | 2006 | IDFG |
|  |  | Capehorn Creek | Sp/Su | W | Juvenile | 2006 | CRITFC |
|  |  | Catherine Creek | Sp/Su | W | Adult | 2002, 2003 | CRITFC |
|  |  | Chamberlin Creek | Sp | W | Juvenile | 2006 | IDFG |
|  |  | Crooked Fork Creek | Sp/Su | W | Juvenile | 2005, 2006 | CRITFC |
|  |  | Dworshak H. | Sp/Su | H | Adult | 2005 | CRITFC |
|  |  | EF Salmon River |  | W | Adult | 2004, 2005 | IDFG |
|  |  | Imnaha R. | Sp/Su | W |  | 1998, 2002, 2003 | CRITFC |
|  |  | Johnson Creek | Sp/Su | W | Adult | 2002, 2003 | CRITFC |
|  |  |  | Sp/Su | H | Juvenile | 2002 - 2004 | CRITFC |
|  |  | Lochsa River (Powell Satellite) | Sp/Su | H | Adult | 2005 | CRITFC |
|  |  | Lolo Creek | Sp/Su | W | Adult | 2001, 2002 | CRITFC |
|  |  |  | Sp/Su | W | Juvenile | 2001 | CRITFC |
|  |  | Looking Glass H. | Sp/Su | H | Juvenile | 1994, 1995, 1998 | CRITFC |
|  |  | Pahsimeroi River | Sp/Su | W | Adult | 2002 | CRITFC |
|  |  | Minam R. | Sp/Su | W |  | 1994, 2002, 2003 | CRITFC |
|  |  | Rapid River H. | Sp | H |  | 1997, 1999, 2002 | CRITFC |
|  |  | Red River | Sp/Su | H | Adult | 2005 | CRITFC |
|  |  | Sawtooth Hatchery | Sp/Su | H | Adult | 2002, 2003 | CRITFC |
|  |  | Sesech R. | Sp/Su | W |  | 2001, 2002, 2003 | CRITFC |
|  |  | Newsome Creek | Sp/Su | W | Adult | 2001, 2002 | CRITFC |
|  |  | Tucannon | Sp/Su | H/W | Adult | 2003 | WDFW |
|  |  | Wenaha Creek | Sp | W | Juvenile | 2002 | IDFG |
|  |  | WF Yankee Fork^3^ |  | W |  | 2005 | IDFG |
| 19 | Washington Coast | Chehalis River | Fa | W | Adult | 1999 | WDFW |
|  |  | Forks Creek H. | Fa | H | Adult | 2005 | WDFW |
|  |  | Hoh River | Fa | W | Adult | 2004, 2005 | WDFW |
|  |  |  | Sp | W | Adult | 1995 - 1998, 2005, 2006 | WDFW |
|  |  | Hoko H. | Fa | H/W | Adult | 2004 | WDFW |
|  |  |  | Fa | W | Adult | 2006 | WDFW |
|  |  | Humtulips H. | Fa | H | Adult | 1990 | WDFW |
|  |  | Makah NFH | Fa | H | Adult | 2001, 2003 | WDFW |
|  |  | Queets | Fa | W | Adult | 1996, 1997 | WDFW |
|  |  | Quillayute/ Bogachiel | Fa | W | Adult | 1995, 1996 | WDFW |
|  |  | Quinalt River | Fa | W | Adult | 1995, 1997, 1998 | WDFW |
|  |  | Quinalt NFH | Fa | H | Adult | 2001 | WDFW |
|  |  | Sol Duc | Sp | H | Adult | 2003 | WDFW |
| 20 | South Puget Sound | Bear Creek | Su/Fa | W | Adult | 1998, 1999, 2003, 2004 | WDFW |
|  |  | Cedar river | Su/Fa | W | Adult | 1994, 2003, 2004 | WDFW |
|  |  | Clear Creek (Nisqually) ^3^ | Fa | H | Adult | 2005 | WDFW |
|  |  | Grovers Creek H. | Su/Fa | H | Adult | 2004 | WDFW |
|  |  | Hupp Springs H.^3^ | Sp | H | Adult | 2002 | WDFW |
|  |  | Issaquah Creek | Su/Fa | W | Adult | 1999 | WDFW |
|  |  |  | Su/Fa | H | Adult | 2004 | WDFW |
|  |  | Nisqually R | Su/Fa | W | Adult | 1998, 1999, 2000, 2006 | WDFW |
|  |  | Soos Creek | Fa | H | Adult | 1998, 2004 | WDFW |
|  |  | South Prairie Creek^3^ | Fa | W | Adult | 1998, 1999, 2002 | WDFW |
|  |  | University of Washington H. | Su/Fa | H | Adult | 2004 | WDFW |
|  |  | Voights Creek^3^ | Fa | H | Adult | 1998 | WDFW |
|  |  | White River | Sp | H | Adult | 1998, 2002 | WDFW |
|  |  |  |  |  |  |  |  |
| 21 | North Puget Sound | Lower Sauk River | Su | W |  | 1998 | NWFSC |
|  |  | Lower Skagit | Fa | W | Adult | 1998, 2006 | WDFW |
|  |  | Marblemount^3^ | Sp | H |  | 1997 | NWFSC |
|  |  |  | Sp | H |  | 2006 | WDFW |
|  |  |  | Su | H |  | 1997 | NWFSC |
|  |  | NF Nooksack | Sp | H  H/W | Juvenile  Adult | 1998  1999 | WDFW |
|  |  | NF Stilliguamish | Su | H/W | Adult | 1996, 2001 | WDFW |
|  |  | Samish | Fa | H | Adult | 1998 | NWFSC |
|  |  | Skagit | Su | W | Adult | 1994, 1995 | WDFW |
|  |  | Skykomish River | Su | W |  | 2004, 2005 | NWFSC |
|  |  |  | Su | W | Adult | 1996, 2000 | WDFW |
|  |  | Snoqualmie |  | W |  | 2005 | NWFSC |
|  |  | Stilliguamish | Su | H | Adult | 2004 | NWFSC |
|  |  | Suiattle (Skagit) | Sp | W | Adult | 1989, 1998, 1999 | WDFW |
|  |  | Suiattle River | Sp | W | Adult | 1998 | NWFSC |
|  |  | Upper Cascade River | Sp | W |  | 1998 | NWFSC |
|  |  |  | Sp | W | Adult | 1998, 1999 | WDFW |
|  |  | Upper Sauk River | Sp | W |  | 1998 | NWFSC |
|  |  | Upper Sauk River | Sp/Su | W | Adult | 1994, 1998, 1999, 2006 | WDFW |
|  |  | Upper Skagit^3^ | Su | W |  | 1998 | NWFSC |
|  |  |  | Su | H | Adult | 1998 | WDFW |
|  |  | Wallace | Su | H |  | 2004, 2005 | NWFSC |
|  |  |  | Su | H | Adult | 1996 | WDFW |
| 22 | Lower Fraser River | Birkenhead River | Sp | H | Adult | 1996, 1997, 1999, 2001 - 2003 | SWFSC |
|  |  | W Chilliwack | Fa | H | Adult | 1998, 1999 | DFO |
|  |  | Maria Slough | Su | W | Adult | 1999 - 2001 | DFO |
| 23 | Lower Thompson River | Nicola | Sp | H |  | 1998, 1999 | OSU |
|  |  | Spius River | Sp | H | Adult | 1996 - 1998 | SWFSC |
| 24 | South Thompson River | Lower Adams | Fa | H | Adult | 1996 | DFO |
|  |  | Lower Thompson | Fa | W | Adult | 2001 | DFO |
|  |  | Middle Shuswap | Fa | H | Adult | 1997 | DFO |
| 25 | North Thompson River | Clearwater | Fa | W | Adult | 1997 | DFO |
|  |  | Deadman^3^ | Sp | H | Adult | 1996 - 1999 | DFO |
|  |  | Louis River | Fa | W | Adult | 2001 | DFO |
|  |  | Raft^3^ | Su | W | Adult | 2001, 2002 | DFO |
| 26 | Mid Fraser River | Chilko | Fa | W | Adult | 1995, 1996, 1999, 2002 | DFO |
|  |  | Nechako | Fa | W | Adult | 1996 | DFO |
|  |  | Quesnel | Fa | W | Adult | 1996 | DFO |
|  |  | Stuart | Fa | W | Adult | 1996 | DFO |
|  |  | Upper Chilcotin | Fa | W | Adult | 2001 | DFO |
| 27 | Upper Fraser River | Morkill River | Fa | W | Adult | 2001 | DFO |
|  |  | Salmon River (Fraser) | Sp | W | Adult | 1997 | SWFSC |
|  |  | Swift | Fa | W | Adult | 1996 | DFO |
|  |  | Torpy River | Fa | W | Adult | 2001 | DFO |
| 28 | East Vancouver Island | Big Qualicum | Fa | H | Adult | 1996 | DFO |
|  |  | Quinsam | Fa | H | Adult | 1996, 1998 | DFO |
|  |  | Cowichan | Fa | H | Adult | 1999, 2000 | DFO |
|  |  | Nanaimo | Fa | H | Adult | 1998, 2002 | DFO |
|  |  | Puntledge | Fa | H | Adult | 2000, 2001 | DFO |
| 29 | West Vancouver Island | Conuma | Fa | H | Adult | 1997 | DFO |
|  |  | Marble at NVI | Fa | H | Adult | 1996, 1999, 2000 | DFO |
|  |  | Nitinat | Fa | H | Adult | 1996 | DFO |
|  |  | Robertson | Fa | H | Adult | 1996, 2003 | DFO |
|  |  | Sarita | Fa | H | Adult | 1997, 2001 | DFO |
|  |  | Tahsis | Fa | W | Adult | 1996, 2002, 2003 | DFO |
|  |  | Tranquil | Fa | W | Adult | 1996, 1999 | DFO |
| 30 | S BC Mainland | Klinaklini | Fa | W | Adult | 1997 | DFO |
|  |  | Porteau  Cove | Fa | H | Adult | 2003 | DFO |
| 31 | Central BC Coast | Atnarko | Fa | H | Adult | 1996 | DFO |
|  |  | Kitimat | Fa | H | Adult | 1997 | DFO |
|  |  | Wannock | Fa | H | Adult | 1996 | DFO |
| 32 | Lower Skeena River | Ecstall | Fa | W | Adult | 2000- 2002 | DFO |
|  |  | Lower Kalum | Fa | W | Adult | 2001 | DFO |
| 33 | Upper Skeena River | Babine | Fa | H | Adult | 1996 | DFO |
|  |  | Bulkley | Fa | W | Adult | 1999 | DFO |
|  |  | Sustut | Fa | W | Adult | 2001 | DFO |
| 34 | Nass River | Damdochax | Fa | W | Adult | 1996 | DFO |
|  |  | Kincolith | Fa | W | Adult | 1996 | DFO |
|  |  | Kwinageese | Fa | W | Adult | 1996 | DFO |
|  |  | Owegee | Fa | W | Adult | 1996 | DFO |
| 35 | Upper Stikine River | Little Tahltan River | Sp | W | Adult | 1989, 1990 | OSU |
| 36 | Taku River | Kowatua Creek |  | W | Adult | 1989, 1990 | ADFG |
|  |  | Nakina River |  | W | Adult | 1989, 1990 | ADFG |
|  |  | Tatsatua Creek |  |  | Adult | 1989, 1990 | ADFG |
|  |  | Upper Nahlin River |  | W | Adult | 1989, 1990, 2004 | ADFG |
| 37 | Southern Southeast Alaska | Chikamin River (West Behm Canal) |  | W | Adult | 1990, 1993 | ADFG |
|  |  | Chikamin River Whitman Lake H. |  | H |  | 2005 | ADFG |
|  |  | Clear Creek |  | W | Adult | 1989, 2003, 2004 | ADFG |
|  |  | Cripple Creek |  | W | Adult | 1988, 2003 | ADFG |
|  |  | Keta River |  | W | Adult | 1989, 2003 | ADFG |
|  |  | King Creek |  | W | Adult | 2003 | ADFG |
| 38 | Southeast Alaska Stikine R. | Andrew Creek, Crystal Lake H. |  | H |  | 2005 | ADFG |
|  |  | Andrew Creek, Macaulay H. |  | H |  | 2005 | ADFG |
|  |  | Andrew Creek, Medvejie H. |  | H |  | 2005 | ADFG |
|  |  | Andrews Creek |  | W | Adult | 1989, 2004 | ADFG |
| 39 | N. Southeast Alaska  King Salmon River | King Salmon River |  | W | Adult | 1989, 1990, 1993 | ADFG |
| 40 | N Southeast Alaska  Chilkat River | Big Boulder Creek |  | W | Adult | 1992, 1995, 2004 | ADFG |
|  |  | Tahini River |  | W | Adult | 1992, 2004 | ADFG |
|  |  | Tahini River, Macaulay H. |  | H |  | 2005 | ADFG |
| 41 | Alsek River | Klukshu River |  | W | Adult | 1989, 1990 | ADFG |
| 42 | Situk River | Situk River |  | W | Adult | 1988, 1990, 1991, 1992 | ADFG |
| 43 | Hood Canal^3^ | George Adams H.  Hamma Hamma River | Fa  Fa | H  W | Adult  Adult | 2005  1999 - 2001 | WDFW  WDFW |
|  |  | NF Skokomish River | Fa | W | Adult | 1998 - 2000, 2004 - 2006 | WDFW |
|  |  | SF Skokomish River | Su/Fa | H/W | Adult | 2005 | WDFW |
| 44 | Juan de Fuca^3^ | Dungeness River  Elwha H. | Fa | W | Adult  Adult | 2004  1996 | WDFW |
|  |  |  |  | H/W | Mixed | 2004, 2005 | NWFSC |

^1^ Run time abbreviations: spring (Sp), summer (Su), fall (Fa), unknown (U) and winter (Wi)

^2^ Laboratory abbreviations: OSU, Oregon State University; SWFSC, Southwest Fisheries Science Center – National Marine Fisheries Service; DFO, Department of Fisheries and Oceans Canada; CRITFC, Columbia River Inter-Tribal Fish Commission; ADFG, Alaska Department of Fish & Game; WDFW, Washington Department of Fish & Wildlife.

^3^ Reporting regions additional to those published in Seeb et al. 2007.

H = Hatchery, NFH = National fish Hatchery
