# Supplementary material for: Geo-Referenced, Abundance Calibrated Ocean Distribution of Chinook Salmon (Oncorhynchus tshawytscha) Stocks across the West Coast of North America
Source: PLoS One. 2015 Jul 22;10(7):e0131276. doi: 10.1371/journal.pone.0131276 (PMC4511799; doi:10.1371/journal.pone.0131276)
Supplement: S2 Appendix — Nine area strata sampled consecutively for five months from May–September 2010. Samples from 8,240 legal-sized Chinook salmon were genotyped and matched to standardized microsatellite (n = 3,866, Oregon) or single-nucleotide polymorphism baseline (n = 4,374, California). (DOC) [file pone.0131276.s003.doc]

| Stock composition results, Oregon fishery and microsatellite baseline | | | | | |  |  |  |  |  |  |  |  |  |  |
| --- | --- | --- | --- | --- | --- | --- | --- | --- | --- | --- | --- | --- | --- | --- | --- |
|  | **Northern Oregon** | | |  |  | **Central Oregon** | | |  |  |  | **Klamath, Oregon** | | |  |
|  | May | June | July | Aug | Sept | May | June | July | Aug | Sept | May | June | July | Aug | Sept |
| Alaska | .00 | .00 | .00 | .00 | .00 | .00 | .00 | .00 | .00 | .00 |  | .00 | .00 | .02 | .00 |
| BC Mainland and Vancouver Island | .01 | .00 | .00 | .00 | .05 | .00 | .00 | .00 | .00 | .00 |  | .00 | .00 | .00 | .00 |
| Fraser and Thompson Rivers | .02 | .04 | .05 | .03 | .14 | .01 | .03 | .00 | .01 | .00 |  | .00 | .11 | .00 | .00 |
| Puget Sound | .02 | .06 | .03 | .02 | .05 | .01 | .01 | .00 | .01 | .00 |  | .00 | .00 | .00 | .01 |
| Juan de Fuca | .00 | .00 | .00 | .00 | .00 | .00 | .00 | .00 | .00 | .00 |  | .00 | .00 | .00 | .00 |
|  |  |  |  |  |  |  |  |  |  |  |  |  |  |  |  |
| Washington Coast | .01 | .00 | .00 | .00 | .00 | .01 | .00 | .00 | .00 | .00 |  | .00 | .00 | .00 | .00 |
| Snake R fall | .02 | .04 | .11 | .09 | .00 | .02 | .03 | .01 | .02 | .02 |  | .10 | .00 | .00 | .00 |
| Mid and Upper Columbia R spring | .00 | .00 | .00 | .00 | .00 | .00 | .00 | .00 | .00 | .00 |  | .00 | .00 | .00 | .00 |
| U Columbia R summer/fall | .14 | .08 | .07 | .07 | .05 | .14 | .08 | .12 | .03 | .02 |  | .05 | .11 | .05 | .00 |
| Mid Columbia R tule | .27 | .33 | .30 | .31 | .00 | .13 | .13 | .22 | .08 | .00 |  | .12 | .11 | .00 | .00 |
|  |  |  |  |  |  |  |  |  |  |  |  |  |  |  |  |
| Willamette R | .02 | .01 | .00 | .00 | .05 | .01 | .00 | .01 | .00 | .00 |  | .02 | .00 | .00 | .00 |
| Deschutes R fall | .01 | .01 | .02 | .00 | .05 | .01 | .01 | .00 | .01 | .00 |  | .00 | .11 | .00 | .00 |
| L Columbia R spring | .01 | .00 | .00 | .00 | .05 | .00 | .00 | .00 | .00 | .02 |  | .00 | .00 | .00 | .00 |
| L Columbia R fall | .07 | .12 | .11 | .16 | .00 | .08 | .08 | .06 | .08 | .07 |  | .02 | .22 | .00 | .00 |
| N Oregon Coast | .01 | .00 | .01 | .02 | .18 | .00 | .00 | .01 | .00 | .02 |  | .00 | .00 | .00 | .01 |
|  |  |  |  |  |  |  |  |  |  |  |  |  |  |  |  |
| Mid Oregon Coast | .08 | .10 | .11 | .12 | .09 | .12 | .14 | .16 | .09 | .20 |  | .07 | .00 | .06 | .08 |
| Rogue R | .09 | .03 | .03 | .01 | .00 | .08 | .09 | .03 | .11 | .11 |  | .14 | .22 | .20 | .36 |
| N California S Oregon Coast | .03 | .00 | .01 | .02 | .00 | .01 | .02 | .00 | .02 | .00 |  | .05 | .11 | .09 | .15 |
| Klamath R | .05 | .02 | .02 | .02 | .00 | .05 | .05 | .03 | .12 | .08 |  | .05 | .00 | .27 | .25 |
| California Coast | .01 | .01 | .00 | .01 | .00 | .01 | .01 | .00 | .02 | .00 |  | .02 | .00 | .02 | .02 |
|  |  |  |  |  |  |  |  |  |  |  |  |  |  |  |  |
| Central Valley fall | .16 | .13 | .13 | .12 | .27 | .27 | .30 | .33 | .40 | .48 |  | .33 | .00 | .30 | .09 |
| Central Valley spring | .00 | .00 | .00 | .00 | .00 | .00 | .00 | .00 | .01 | .00 |  | .02 | .00 | .00 | .01 |
| Central Valley winter | .00 | .00 | .00 | .00 | .00 | .00 | .00 | .00 | .00 | .00 |  | .00 | .00 | .00 | .00 |
|  |  |  |  |  |  |  |  |  |  |  |  |  |  |  |  |
|  |  |  |  |  |  |  |  |  |  |  |  |  |  |  |  |
| Stock compositions, CA fishery and SNP baseline | | | |  |  |  |  |  |  |  |  |  |  |  |  |
|  | **Klamath, north, California** | | | | | **Fort Bragg** | |  |  |  | **San Francisco, north** | | | |  |
|  |  | June | July | Aug | Sept | May | June | July | Aug | Sept | May | June | July | Aug | Sept |
| Alaska |  | .00 | .00 | .00 | .00 | .00 | .00 | .00 | .00 | .00 | .00 | .00 | .00 | .00 | .00 |
| BC Mainland and Vancouver Island |  | .00 | .00 | .00 | .00 | .00 | .00 | .00 | .00 | .00 | .00 | .00 | .00 | .00 | .00 |
| Fraser and Thompson Rivers |  | .00 | .00 | .00 | .00 | .00 | .00 | .00 | .00 | .00 | .00 | .00 | .00 | .00 | .00 |
| Puget Sound |  | .00 | .00 | .00 | .00 | .00 | .00 | .00 | .00 | .00 | .00 | .00 | .00 | .00 | .00 |
| Juan de Fuca |  | .00 | .00 | .00 | .00 | .00 | .00 | .00 | .00 | .00 | .00 | .00 | .00 | .00 | .00 |
|  |  |  |  |  |  |  |  |  |  |  |  |  |  |  |  |
| Washington Coast |  | .00 | .00 | .00 | .00 | .00 | .00 | .00 | .00 | .00 | .00 | .00 | .00 | .00 | .00 |
| Snake R fa |  | .00 | .01 | .01 | .00 | .00 | .00 | .00 | .00 | .00 | .00 | .00 | .01 | .00 | .00 |
| Mid and Upper Columbia R sp |  | .00 | .00 | .00 | .00 | .00 | .00 | .00 | .00 | .00 | .00 | .00 | .00 | .00 | .00 |
| U Columbia R sufa |  | .00 | .02 | .00 | .00 | .02 | .00 | .01 | .00 | .00 | .03 | .00 | .00 | .00 | .00 |
| Mid Columbia R tule |  | .02 | .01 | .00 | .00 | .00 | .00 | .00 | .00 | .00 | .00 | .00 | .00 | .00 | .00 |
|  |  |  |  |  |  |  |  |  |  |  |  |  |  |  |  |
| Willamette R |  | .00 | .00 | .00 | .00 | .00 | .00 | .00 | .00 | .00 | .00 | .00 | .00 | .00 | .00 |
| Deschutes R fa |  | .02 | .01 | .01 | .00 | .00 | .00 | .00 | .00 | .00 | .00 | .00 | .00 | .00 | .00 |
| L Columbia R sp |  | .00 | .00 | .00 | .00 | .00 | .00 | .00 | .00 | .00 | .00 | .00 | .00 | .00 | .00 |
| L Columbia R fa |  | .00 | .01 | .00 | .00 | .00 | .00 | .00 | .00 | .00 | .00 | .00 | .00 | .00 | .00 |
| N Oregon Coast |  | .00 | .01 | .00 | .00 | .00 | .00 | .00 | .00 | .00 | .00 | .00 | .00 | .00 | .00 |
|  |  |  |  |  |  |  |  |  |  |  |  |  |  |  |  |
| Mid Oregon Coast |  | .02 | .01 | .01 | .01 | .04 | .00 | .02 | .01 | .00 | .06 | .01 | .05 | .00 | .00 |
| Rogue R |  | .09 | .16 | .29 | .34 | .20 | .16 | .22 | .17 | .31 | .28 | .09 | .20 | .00 | .00 |
| N California S Oregon Coast |  | .05 | .09 | .06 | .21 | .09 | .21 | .16 | .09 | .17 | .06 | .08 | .13 | .01 | .00 |
| Klamath R |  | .11 | .17 | .24 | .13 | .37 | .31 | .12 | .08 | .05 | .25 | .07 | .16 | .01 | .00 |
| California Coast |  | .02 | .09 | .05 | .15 | .10 | .13 | .11 | .14 | .28 | .03 | .06 | .15 | .02 | .03 |
|  |  |  |  |  |  |  |  |  |  |  |  |  |  |  |  |
| Central Valley fa |  | .69 | .42 | .32 | .16 | .18 | .18 | .35 | .50 | .18 | .31 | .62 | .29 | .94 | .92 |
| Central Valley sp |  | .00 | .02 | .01 | .00 | .00 | .00 | .00 | .01 | .02 | .00 | .07 | .01 | .01 | .05 |
| Central Valley wi |  | .00 | .00 | .00 | .00 | .00 | .00 | .00 | .00 | .00 | .00 | .00 | .00 | .00 | .00 |
|  |  |  |  |  |  |  |  |  |  |  |  |  |  |  |  |
|  |  |  |  |  |  |  |  |  |  |  |  |  |  |  |  |
|  | **San Francisco, south** | | | |  | **Monterey Bay, north** | | | |  | **Monterey Bay, south** | | | |  |
|  | May | June | July | Aug | Sept | May | June | July | Aug | Sept | May | June | July | Aug | Sept |
| Alaska | .00 | .00 | .00 | .00 | .00 | .00 | .00 | .00 | .00 | .00 | .00 | .00 | .00 | .00 | .00 |
| BC Mainland and Vancouver Island | .00 | .01 | .00 | .00 | .00 | .00 | .00 | .00 | .01 | .00 | .00 | .00 | .00 | .00 | .00 |
| Fraser and Thompson Rivers | .00 | .00 | .00 | .00 | .00 | .00 | .00 | .00 | .00 | .00 | .00 | .00 | .00 | .00 | .00 |
| Puget Sound | .00 | .00 | .00 | .00 | .00 | .00 | .00 | .00 | .00 | .00 | .00 | .00 | .00 | .00 | .00 |
| Juan de Fuca | .00 | .00 | .00 | .00 | .00 | .00 | .00 | .00 | .00 | .00 | .00 | .00 | .00 | .00 | .00 |
|  |  |  |  |  |  |  |  |  |  |  |  |  |  |  |  |
| Washington Coast | .00 | .00 | .00 | .00 | .00 | .00 | .00 | .00 | .00 | .00 | .00 | .00 | .00 | .00 | .00 |
| Snake R fa | .00 | .00 | .00 | .00 | .00 | .00 | .00 | .00 | .00 | .00 | .00 | .00 | .00 | .00 | .00 |
| Mid and Upper Columbia R sp | .00 | .00 | .00 | .00 | .00 | .00 | .00 | .00 | .00 | .00 | .00 | .00 | .00 | .00 | .00 |
| U Columbia R sufa | .01 | .01 | .00 | .00 | .00 | .00 | .00 | .00 | .00 | .00 | .00 | .00 | .00 | .00 | .00 |
| Mid Columbia R tule | .00 | .00 | .00 | .00 | .00 | .00 | .00 | .00 | .00 | .00 | .00 | .00 | .00 | .00 | .00 |
|  |  |  |  |  |  |  |  |  |  |  |  |  |  |  |  |
| Willamette R | .00 | .00 | .00 | .00 | .00 | .00 | .00 | .00 | .00 | .00 | .00 | .00 | .00 | .00 | .00 |
| Deschutes R fa | .00 | .00 | .00 | .00 | .00 | .00 | .00 | .00 | .00 | .00 | .00 | .00 | .00 | .00 | .00 |
| L Columbia R sp | .01 | .00 | .00 | .00 | .00 | .00 | .00 | .00 | .00 | .00 | .00 | .00 | .00 | .00 | .00 |
| L Columbia R fa | .00 | .00 | .00 | .00 | .00 | .00 | .00 | .00 | .00 | .00 | .00 | .00 | .00 | .00 | .00 |
| N Oregon Coast | .00 | .00 | .00 | .00 | .00 | .00 | .00 | .00 | .01 | .00 | .00 | .00 | .00 | .00 | .00 |
|  |  |  |  |  |  |  |  |  |  |  |  |  |  |  |  |
| Mid Oregon Coast | .03 | .02 | .04 | .00 | .00 | .00 | .00 | .00 | .00 | .00 | .00 | .00 | .00 | .00 | .00 |
| Rogue R | .00 | .03 | .03 | .00 | .01 | .09 | .00 | .01 | .00 | .02 | .00 | .00 | .17 | .00 | .06 |
| N California S Oregon Coast | .09 | .01 | .02 | .00 | .00 | .00 | .00 | .01 | .01 | .00 | .00 | .00 | .08 | .00 | .00 |
| Klamath R | .01 | .03 | .03 | .01 | .00 | .00 | .06 | .00 | .00 | .00 | .00 | .00 | .00 | .00 | .00 |
| California Coast | .02 | .01 | .06 | .00 | .03 | .09 | .00 | .04 | .04 | .02 | .00 | .00 | .08 | .11 | .00 |
|  |  |  |  |  |  |  |  |  |  |  |  |  |  |  |  |
| Central Valley fa | .76 | .85 | .80 | .98 | .91 | .82 | .88 | .93 | .90 | .76 | 1.00 | 1.00 | .67 | .11 | .31 |
| Central Valley sp | .06 | .04 | .01 | .01 | .04 | .00 | .06 | .01 | .01 | .14 | .00 | .00 | .00 | .00 | .06 |
| Central Valley wi | .00 | .01 | .00 | .00 | .00 | .00 | .00 | .00 | .03 | .06 | .00 | .00 | .00 | .78 | .56 |
