# Supplementary material for: Geo-Referenced, Abundance Calibrated Ocean Distribution of Chinook Salmon (Oncorhynchus tshawytscha) Stocks across the West Coast of North America
Source: PLoS One. 2015 Jul 22;10(7):e0131276. doi: 10.1371/journal.pone.0131276 (PMC4511799; doi:10.1371/journal.pone.0131276)
Supplement: S3 Appendix — Fish were sampled during open commercial fisheries or from closed areas using non-retention sampling techniques. (DOC) [file pone.0131276.s004.doc]

Supplemental Appendix 3. Stock-specific catch per unit effort (CPUE) for the 2010 at-sea Oregon and California Chinook salmon troll fishery calculated for nine areas from stock composition results (Supplemental Appendix 4) and CPUE (Table 3).

| Genetic Region | North Oregon Coast | | | | | | Central Oregon Coast | | | | | | | Klamath Zone – OR | | | | |
| --- | --- | --- | --- | --- | --- | --- | --- | --- | --- | --- | --- | --- | --- | --- | --- | --- | --- | --- |
|  | May | June | July | Aug | Sept | May | | June | July | Aug | Sept | May | June | | July | Aug | Sept |  |
| Alaska | 0.01 | 0.01 | 0.00 | 0.00 | 0.00 | 0.02 | | 0.00 | 0.00 | 0.00 | 0.00 | 0.00 | 0.00 | | 0.00 | 0.04 | 0.00 |  |
| BC Mainland and Vancouver Island | 0.04 | 0.02 | 0.01 | 0.01 | 0.03 | 0.00 | | 0.00 | 0.00 | 0.01 | 0.00 | 0.00 | 0.00 | | 0.00 | 0.00 | 0.00 |  |
| Fraser and Thompson Rivers | 0.10 | 0.25 | 0.28 | 0.10 | 0.10 | 0.05 | | 0.11 | 0.00 | 0.04 | 0.00 | 0.00 | 0.00 | | 0.16 | 0.00 | 0.00 |  |
| Puget Sound | 0.10 | 0.38 | 0.18 | 0.05 | 0.03 | 0.04 | | 0.05 | 0.00 | 0.02 | 0.00 | 0.00 | 0.00 | | 0.00 | 0.00 | 0.05 |  |
| Juan de Fuca | 0.00 | 0.00 | 0.00 | 0.00 | 0.00 | 0.00 | | 0.00 | 0.00 | 0.00 | 0.00 | 0.00 | 0.00 | | 0.00 | 0.00 | 0.00 |  |
| Washington Coast | 0.03 | 0.01 | 0.00 | 0.00 | 0.00 | 0.03 | | 0.01 | 0.00 | 0.00 | 0.00 | 0.00 | 0.00 | | 0.00 | 0.00 | 0.00 |  |
| Snake R fall | 0.11 | 0.28 | 0.61 | 0.26 | 0.00 | 0.09 | | 0.10 | 0.03 | 0.05 | 0.03 | 0.00 | 0.12 | | 0.00 | 0.00 | 0.00 |  |
| Snake R spring summer | 0.00 | 0.01 | 0.00 | 0.00 | 0.03 | 0.01 | | 0.00 | 0.00 | 0.00 | 0.00 | 0.00 | 0.00 | | 0.00 | 0.00 | 0.00 |  |
| Mid and Upper Columbia R spring | 0.00 | 0.00 | 0.00 | 0.00 | 0.00 | 0.01 | | 0.00 | 0.00 | 0.00 | 0.00 | 0.00 | 0.00 | | 0.00 | 0.00 | 0.00 |  |
| U Columbia R summer/fall | 0.73 | 0.51 | 0.40 | 0.21 | 0.03 | 0.60 | | 0.31 | 0.26 | 0.08 | 0.03 | 0.00 | 0.06 | | 0.16 | 0.12 | 0.00 |  |
| Mid Columbia R tule | 1.46 | 2.08 | 1.62 | 0.90 | 0.00 | 0.55 | | 0.48 | 0.49 | 0.22 | 0.00 | 0.00 | 0.15 | | 0.16 | 0.00 | 0.00 |  |
| Willamette R | 0.10 | 0.03 | 0.00 | 0.01 | 0.03 | 0.06 | | 0.01 | 0.03 | 0.00 | 0.00 | 0.00 | 0.03 | | 0.00 | 0.00 | 0.00 |  |
| Deschutes R fall | 0.06 | 0.05 | 0.10 | 0.01 | 0.03 | 0.06 | | 0.05 | 0.00 | 0.04 | 0.00 | 0.00 | 0.00 | | 0.16 | 0.00 | 0.00 |  |
| L Columbia R spring | 0.03 | 0.02 | 0.00 | 0.00 | 0.03 | 0.00 | | 0.01 | 0.00 | 0.00 | 0.03 | 0.00 | 0.00 | | 0.00 | 0.00 | 0.00 |  |
| L Columbia R fall | 0.37 | 0.73 | 0.58 | 0.45 | 0.00 | 0.33 | | 0.31 | 0.13 | 0.25 | 0.14 | 0.00 | 0.03 | | 0.32 | 0.00 | 0.00 |  |
| N Oregon Coast | 0.06 | 0.02 | 0.03 | 0.07 | 0.13 | 0.02 | | 0.01 | 0.03 | 0.01 | 0.03 | 0.00 | 0.00 | | 0.00 | 0.00 | 0.05 |  |
| Mid Oregon Coast | 0.41 | 0.64 | 0.61 | 0.34 | 0.06 | 0.50 | | 0.52 | 0.36 | 0.28 | 0.42 | 0.00 | 0.09 | | 0.00 | 0.15 | 0.34 |  |
| Rogue R | 0.46 | 0.19 | 0.18 | 0.04 | 0.00 | 0.35 | | 0.32 | 0.07 | 0.31 | 0.24 | 0.00 | 0.18 | | 0.32 | 0.50 | 1.50 |  |
| N California S Oregon Coast | 0.15 | 0.02 | 0.03 | 0.05 | 0.00 | 0.06 | | 0.07 | 0.00 | 0.06 | 0.00 | 0.00 | 0.06 | | 0.16 | 0.23 | 0.63 |  |
| Klamath R | 0.25 | 0.12 | 0.13 | 0.05 | 0.00 | 0.22 | | 0.18 | 0.07 | 0.35 | 0.17 | 0.00 | 0.06 | | 0.00 | 0.70 | 1.02 |  |
| California Coast | 0.03 | 0.03 | 0.00 | 0.03 | 0.00 | 0.05 | | 0.05 | 0.00 | 0.05 | 0.00 | 0.00 | 0.03 | | 0.00 | 0.04 | 0.10 |  |
| Central Valley fall | 0.87 | 0.82 | 0.70 | 0.34 | 0.19 | 1.12 | | 1.12 | 0.72 | 1.16 | 1.01 | 0.00 | 0.42 | | 0.00 | 0.77 | 0.39 |  |
| Central Valley spring | 0.00 | 0.01 | 0.00 | 0.00 | 0.00 | 0.01 | | 0.01 | 0.00 | 0.02 | 0.00 | 0.00 | 0.03 | | 0.00 | 0.00 | 0.05 |  |
| Central Valley winter | 0.00 | 0.00 | 0.00 | 0.00 | 0.00 | 0.00 | | 0.00 | 0.00 | 0.00 | 0.00 | 0.00 | 0.00 | | 0.00 | 0.00 | 0.00 |  |
|  |  |  |  |  |  |  | |  |  |  |  |  |  | |  |  |  |  |
| Sum CPUE | 5.37 | 6.23 | 5.46 | 2.89 | 0.70 | 4.17 | | 3.71 | 2.20 | 2.94 | 2.13 | 0.00 | 1.26 | | 1.43 | 2.56 | 4.11 |  |

| Genetic Region | Klamath Zone, CA | | | | Fort Bragg | | | | | San Francisco, North | | | | |
| --- | --- | --- | --- | --- | --- | --- | --- | --- | --- | --- | --- | --- | --- | --- |
|  | June | July | Aug | Sept | May | June | July | Aug | Sept | May | June | July | Aug | Sept |
| Alaska | 0.00 | 0.00 | 0.00 | 0.00 | 0.00 | 0.00 | 0.00 | 0.00 | 0.00 | 0.00 | 0.00 | 0.00 | 0.00 | 0.00 |
| BC Mainland and Vancouver Island | 0.00 | 0.00 | 0.00 | 0.00 | 0.00 | 0.00 | 0.00 | 0.00 | 0.00 | 0.00 | 0.00 | 0.00 | 0.00 | 0.00 |
| Fraser and Thompson Rivers | 0.00 | 0.00 | 0.00 | 0.00 | 0.00 | 0.00 | 0.00 | 0.00 | 0.00 | 0.00 | 0.00 | 0.00 | 0.00 | 0.00 |
| Puget Sound | 0.00 | 0.00 | 0.00 | 0.00 | 0.00 | 0.00 | 0.00 | 0.00 | 0.01 | 0.00 | 0.00 | 0.00 | 0.00 | 0.00 |
| Juan de Fuca | 0.00 | 0.00 | 0.00 | 0.00 | 0.00 | 0.00 | 0.00 | 0.00 | 0.00 | 0.00 | 0.00 | 0.00 | 0.00 | 0.00 |
| Washington Coast | 0.00 | 0.00 | 0.00 | 0.00 | 0.00 | 0.00 | 0.00 | 0.00 | 0.00 | 0.00 | 0.00 | 0.00 | 0.00 | 0.00 |
| Snake R fa | 0.00 | 0.02 | 0.05 | 0.00 | 0.00 | 0.00 | 0.01 | 0.00 | 0.00 | 0.00 | 0.00 | 0.03 | 0.00 | 0.00 |
| Snake R spring summer | 0.00 | 0.00 | 0.00 | 0.00 | 0.00 | 0.00 | 0.00 | 0.00 | 0.00 | 0.00 | 0.00 | 0.00 | 0.00 | 0.00 |
| Mid and Upper Columbia R sp | 0.00 | 0.00 | 0.00 | 0.00 | 0.00 | 0.00 | 0.00 | 0.00 | 0.00 | 0.00 | 0.00 | 0.00 | 0.00 | 0.00 |
| U Columbia R sufa | 0.00 | 0.04 | 0.00 | 0.00 | 0.22 | 0.00 | 0.03 | 0.00 | 0.00 | 0.04 | 0.00 | 0.00 | 0.00 | 0.00 |
| Mid Columbia R tule | 0.03 | 0.02 | 0.02 | 0.00 | 0.00 | 0.00 | 0.00 | 0.00 | 0.00 | 0.00 | 0.00 | 0.01 | 0.00 | 0.00 |
| Willamette R | 0.00 | 0.00 | 0.00 | 0.00 | 0.00 | 0.00 | 0.00 | 0.00 | 0.00 | 0.00 | 0.00 | 0.00 | 0.00 | 0.00 |
| Deschutes R fa | 0.03 | 0.02 | 0.03 | 0.00 | 0.00 | 0.00 | 0.02 | 0.00 | 0.01 | 0.00 | 0.00 | 0.00 | 0.00 | 0.00 |
| L Columbia R sp | 0.00 | 0.00 | 0.00 | 0.00 | 0.00 | 0.00 | 0.00 | 0.00 | 0.00 | 0.00 | 0.00 | 0.00 | 0.00 | 0.00 |
| L Columbia R fa | 0.00 | 0.02 | 0.00 | 0.00 | 0.00 | 0.00 | 0.01 | 0.00 | 0.00 | 0.00 | 0.00 | 0.00 | 0.00 | 0.00 |
| N Oregon Coast | 0.00 | 0.02 | 0.00 | 0.00 | 0.00 | 0.00 | 0.00 | 0.00 | 0.01 | 0.00 | 0.00 | 0.00 | 0.00 | 0.00 |
| Mid Oregon Coast | 0.03 | 0.02 | 0.05 | 0.05 | 0.44 | 0.00 | 0.09 | 0.05 | 0.00 | 0.09 | 0.02 | 0.21 | 0.00 | 0.00 |
| Rogue R | 0.16 | 0.36 | 1.84 | 1.39 | 2.00 | 0.54 | 1.17 | 0.77 | 1.92 | 0.43 | 0.14 | 0.84 | 0.00 | 0.00 |
| N California S Oregon Coast | 0.08 | 0.20 | 0.40 | 0.85 | 0.89 | 0.72 | 0.84 | 0.39 | 1.05 | 0.09 | 0.12 | 0.55 | 0.03 | 0.00 |
| Klamath R | 0.18 | 0.40 | 1.54 | 0.52 | 3.78 | 1.06 | 0.66 | 0.35 | 0.29 | 0.39 | 0.10 | 0.70 | 0.02 | 0.00 |
| California Coast | 0.03 | 0.20 | 0.30 | 0.64 | 1.00 | 0.46 | 0.59 | 0.60 | 1.74 | 0.04 | 0.09 | 0.63 | 0.05 | 0.02 |
| Central Valley fa | 1.16 | 0.96 | 2.04 | 0.65 | 1.78 | 0.61 | 1.82 | 2.24 | 1.16 | 0.47 | 0.91 | 1.26 | 2.17 | 0.57 |
| Central Valley sp | 0.00 | 0.04 | 0.08 | 0.02 | 0.00 | 0.00 | 0.02 | 0.03 | 0.10 | 0.00 | 0.10 | 0.05 | 0.03 | 0.03 |
| Central Valley wi | 0.00 | 0.00 | 0.00 | 0.00 | 0.00 | 0.00 | 0.00 | 0.00 | 0.00 | 0.00 | 0.00 | 0.00 | 0.00 | 0.00 |
| Sum CPUE | 1.68 | 2.31 | 6.37 | 4.12 | 10.11 | 3.38 | 5.27 | 4.44 | 6.30 | 1.54 | 1.47 | 4.28 | 2.30 | 0.62 |

| Genetic Region | San Francisco, north | | | | | Monterey, north | | | | | Monterey, south | | | | |
| --- | --- | --- | --- | --- | --- | --- | --- | --- | --- | --- | --- | --- | --- | --- | --- |
|  | May | June | July | Aug | Sept | May | June | July | Aug | Sept | May | June | July | Aug | Sept |
| Alaska | 0.00 | 0.00 | 0.00 | 0.00 | 0.00 | 0.00 | 0.00 | 0.01 | 0.00 | 0.00 | 0.00 | 0.00 | 0.00 | 0.00 | 0.00 |
| BC Mainland and Vancouver Island | 0.00 | 0.02 | 0.00 | 0.00 | 0.00 | 0.00 | 0.00 | 0.00 | 0.03 | 0.00 | 0.00 | 0.00 | 0.00 | 0.00 | 0.00 |
| Fraser and Thompson Rivers | 0.00 | 0.00 | 0.00 | 0.00 | 0.00 | 0.00 | 0.00 | 0.00 | 0.00 | 0.00 | 0.00 | 0.00 | 0.00 | 0.00 | 0.00 |
| Puget Sound | 0.00 | 0.00 | 0.00 | 0.00 | 0.00 | 0.00 | 0.00 | 0.00 | 0.00 | 0.00 | 0.00 | 0.00 | 0.00 | 0.00 | 0.00 |
| Juan de Fuca | 0.00 | 0.00 | 0.00 | 0.00 | 0.00 | 0.00 | 0.00 | 0.00 | 0.00 | 0.00 | 0.00 | 0.00 | 0.00 | 0.00 | 0.00 |
| Washington Coast | 0.00 | 0.00 | 0.00 | 0.00 | 0.00 | 0.00 | 0.00 | 0.00 | 0.00 | 0.00 | 0.00 | 0.00 | 0.00 | 0.00 | 0.00 |
| Snake R fa | 0.00 | 0.00 | 0.00 | 0.00 | 0.00 | 0.00 | 0.00 | 0.00 | 0.00 | 0.00 | 0.00 | 0.00 | 0.00 | 0.00 | 0.00 |
| Snake R spring summer | 0.00 | 0.00 | 0.00 | 0.00 | 0.00 | 0.00 | 0.00 | 0.00 | 0.00 | 0.00 | 0.00 | 0.00 | 0.00 | 0.00 | 0.00 |
| Mid and Upper Columbia R sp | 0.00 | 0.00 | 0.00 | 0.00 | 0.00 | 0.00 | 0.00 | 0.00 | 0.00 | 0.00 | 0.00 | 0.00 | 0.00 | 0.00 | 0.00 |
| U Columbia R sufa | 0.02 | 0.02 | 0.00 | 0.00 | 0.00 | 0.00 | 0.00 | 0.00 | 0.00 | 0.00 | 0.00 | 0.00 | 0.00 | 0.00 | 0.00 |
| Mid Columbia R tule | 0.00 | 0.00 | 0.00 | 0.00 | 0.00 | 0.00 | 0.00 | 0.00 | 0.00 | 0.00 | 0.00 | 0.00 | 0.00 | 0.00 | 0.00 |
| Willamette R | 0.00 | 0.00 | 0.00 | 0.00 | 0.00 | 0.00 | 0.00 | 0.00 | 0.00 | 0.00 | 0.00 | 0.00 | 0.00 | 0.00 | 0.00 |
| Deschutes R fa | 0.00 | 0.00 | 0.00 | 0.00 | 0.00 | 0.00 | 0.00 | 0.00 | 0.00 | 0.00 | 0.00 | 0.00 | 0.00 | 0.00 | 0.00 |
| L Columbia R sp | 0.02 | 0.00 | 0.00 | 0.00 | 0.00 | 0.00 | 0.00 | 0.00 | 0.00 | 0.00 | 0.00 | 0.00 | 0.00 | 0.00 | 0.00 |
| L Columbia R fa | 0.00 | 0.00 | 0.00 | 0.00 | 0.00 | 0.00 | 0.00 | 0.00 | 0.00 | 0.00 | 0.00 | 0.00 | 0.00 | 0.00 | 0.00 |
| N Oregon Coast | 0.00 | 0.00 | 0.00 | 0.00 | 0.00 | 0.00 | 0.00 | 0.00 | 0.03 | 0.00 | 0.00 | 0.00 | 0.00 | 0.00 | 0.00 |
| Mid Oregon Coast | 0.06 | 0.05 | 0.05 | 0.00 | 0.00 | 0.00 | 0.00 | 0.01 | 0.00 | 0.00 | 0.00 | 0.00 | 0.00 | 0.00 | 0.00 |
| Rogue R | 0.00 | 0.09 | 0.04 | 0.00 | 0.01 | 0.03 | 0.00 | 0.02 | 0.00 | 0.04 | 0.00 | 0.00 | 0.07 | 0.00 | 0.08 |
| N California S Oregon Coast | 0.15 | 0.02 | 0.02 | 0.00 | 0.00 | 0.00 | 0.00 | 0.04 | 0.03 | 0.00 | 0.00 | 0.00 | 0.04 | 0.00 | 0.00 |
| Klamath R | 0.02 | 0.09 | 0.04 | 0.02 | 0.00 | 0.00 | 0.02 | 0.01 | 0.00 | 0.00 | 0.00 | 0.00 | 0.00 | 0.00 | 0.00 |
| California Coast | 0.04 | 0.03 | 0.07 | 0.00 | 0.03 | 0.03 | 0.00 | 0.18 | 0.10 | 0.04 | 0.00 | 0.00 | 0.04 | 0.06 | 0.00 |
| Central Valley fa | 1.25 | 2.82 | 0.92 | 1.93 | 0.95 | 0.25 | 0.36 | 4.26 | 2.58 | 1.36 | 0.24 | 0.34 | 0.30 | 0.06 | 0.42 |
| Central Valley sp | 0.10 | 0.14 | 0.01 | 0.02 | 0.04 | 0.00 | 0.02 | 0.05 | 0.03 | 0.25 | 0.00 | 0.00 | 0.00 | 0.00 | 0.08 |
| Central Valley wi | 0.00 | 0.03 | 0.00 | 0.00 | 0.00 | 0.00 | 0.00 | 0.00 | 0.08 | 0.11 | 0.00 | 0.00 | 0.00 | 0.41 | 0.75 |
| Sum CPUE | 1.65 | 3.30 | 1.15 | 1.97 | 1.04 | 0.31 | 0.40 | 4.60 | 2.85 | 1.79 | 0.24 | 0.34 | 0.45 | 0.53 | 1.33 |
