# Supplementary material for: Geo-Referenced, Abundance Calibrated Ocean Distribution of Chinook Salmon (Oncorhynchus tshawytscha) Stocks across the West Coast of North America
Source: PLoS One. 2015 Jul 22;10(7):e0131276. doi: 10.1371/journal.pone.0131276 (PMC4511799; doi:10.1371/journal.pone.0131276)
Supplement: S4 Appendix — (DOC) [file pone.0131276.s005.doc]

Supplemental Appendix 4. Genetic Stock Identification (GSI) individual assignment and Coded-Wire-Tag stock origin evaluation.

| Barcode | Snout ID | Chinook Run | CWT Release Date | Release Site | Hatchery | Stock | Release Agency | CWT Recovery Date | **GSI Posterior Probabilty** | GSI Stock assignment | GSI Correct? |
| --- | --- | --- | --- | --- | --- | --- | --- | --- | --- | --- | --- |
| 35413 | 10J6147 | Fall | 09-May-08 | San Pablo Bay Net Pen | Mokelumne R Fish Ins | American R | CDFG | 03-Aug-10 | **1.00** | Central Valley fall | **yes** |
| 29012 | 10J7709 | Fall | 09-May-08 | San Pablo Bay Net Pen | Mokelumne R Fish Ins | American R | CDFG | 26-Jul-10 | **0.99** | Central Valley fall | **yes** |
| 18398 | 10J7754 | Fall | 09-May-08 | San Pablo Bay Net Pen | Mokelumne R Fish Ins | American R | CDFG | 02-Aug-10 | **1.00** | Central Valley fall | **yes** |
| 35940 | 10J6160 | Fall | 01-Sep-08 | Tiburon Net Pens | Mokelumne R Fish Ins | American R | CDFG | 09-Aug-10 | **1.00** | Central Valley fall | **yes** |
| 30090 | 10J7760 | Fall | 01-Sep-08 | Tiburon Net Pens | Mokelumne R Fish Ins | American R | CDFG | 12-Aug-10 | **1.00** | Central Valley fall | **yes** |
| 35881 | 10J5546 | Fall | 21-May-08 | West Sacramento | Feather R H | Feather R | FWS | 18-Aug-10 | **1.00** | Central Valley fall | **yes** |
| 35921 | 10J6159 | Spring | 07-Apr-08 | San Pablo Bay Net Pen | Feather R H | Feather R | CDWR | 09-Aug-10 | **1.00** | Central Valley fall | **yes** |
| 35393 | 10J6143 | Fall | 12-Jun-07 | Wickland Oil Terminal | Feather R H | Feather R | CDFG | 30-Jun-10 | **1.00** | Central Valley fall | **yes** |
| 33223 | 10J3000 | Fall | 25-Apr-08 | Mare Island Net Pen | Feather R H | Feather R | CDFG | 30-Jun-10 | **1.00** | Central Valley fall | **yes** |
| 35870 | 10J5547 | Fall | 25-Apr-08 | Mare Island Net Pen | Feather R H | Feather R | CDFG | 18-Aug-10 | **1.00** | Central Valley fall | **yes** |
| 33124 | 10J3204 | Fall | 22-May-08 | Mare Island Net Pen | Feather R H | Feather R | CDFG | 06-Jun-10 | **1.00** | Central Valley fall | **yes** |
| 18478 | 10J3193 | Fall | 08-May-08 | San Pablo Bay Net Pen | Mokelumne R Fish Ins | Mokelumne R | CDFG | 07-Jun-10 | **0.99** | Central Valley fall | **yes** |
| 33185 | 10J3202 | Fall | 08-May-08 | San Pablo Bay Net Pen | Mokelumne R Fish Ins | Mokelumne R | CDFG | 06-Jun-10 | **0.99** | Central Valley fall | **yes** |
| 18395 | 10J3229 | Fall | 08-May-08 | San Pablo Bay Net Pen | Mokelumne R Fish Ins | Mokelumne R | CDFG | 20-Jun-10 | **1.00** | Central Valley fall | **yes** |
| 35883 | 10J5545 | Fall | 08-May-08 | San Pablo Bay Net Pen | Mokelumne R Fish Ins | Mokelumne R | CDFG | 18-Aug-10 | **1.00** | Central Valley fall | **yes** |
| 18784 | 10J6152 | Fall | 08-May-08 | San Pablo Bay Net Pen | Mokelumne R Fish Ins | Mokelumne R | CDFG | 06-Aug-10 | **0.95** | Central Valley fall | **yes** |
| 35426 | 10J6148 | Fall | 19-Jun-06 | Cowlitz R | Cowlitz Salmon H | Cowlitz R | WDFW | 03-Aug-10 | **1.00** | L Columbia fall | **yes** |
| 33437 | 10J2939 | Fall | 20-May-08 | R Chilliwack R | H Chilliwack R | S- Chilliwack R | CDFO | 23-Jun-10 | **1.00** | L Fraser | **yes** |
| 18492 | 10J6151 | Fall | 20-May-08 | R Chilliwack  R | H Chilliwack R | S- Chilliwack K R | CDFO | 05-Aug-10 | **1.00** | L Fraser | **yes** |
| Barcode | Coded-wire-tag ID | Chinook Run | CWT Release Date | Release Site | Hatchery | Stock | Release Agency | CWT Recovery Date | **GSI Posterior Probabilty** | GSI Stock assignment | GSI Correct? |
|  |  |  |  |  |  |  |  |  |  |  |  |
| 32892 | 10J2940 | URB L-Fall | 14-May-08 | Big Creek (L COL R) | Big CR H | Big CR H | ODFW | 23-Jun-10 | **1.00** | Mid Columbia tule | **yes** |
| 18329 | 10J3195 | Fall | 02-May-08 | Spring CR | Spring CR NFH | Spring CR | FWS | 06-Jun-10 | **1.00** | Mid Columbia tule | **yes** |
| 18327 | 10J3196 | Fall | 02-May-08 | Spring CR | Spring CR NFH | Spring CR NFH | FWS | 06-Jun-10 | **1.00** | Mid Columbia tule | **yes** |
| 33520 | 10J7755 | Fall | 10-Apr-08 | Spring CR | Spring CR NFH | Spring CR NFH | FWS | 01-Aug-10 | **0.98** | Mid Columbia tule | **yes** |
| 5121 | 10J7712 | Fall | 10-Apr-08 | Spring CR | Spring CR NFH | Spring CR NFH | FWS | 26-Jul-10 | **1.00** | Mid Columbia tule | **yes** |
| 5415 | 10J2998 | URB L-Fall | 26-May-08 | Big Canyon Accl Pond | Lyons Ferry H | Lyons Ferry H | NEZP | 01-Jul-10 | **0.99** | Snake fall | **yes** |
| 33258 | 10J3316 | URB L-Fall | 28-May-08 | Captain Johns PD | Lyons Ferry H | Lyons Ferry H | NEZP | 19-Jul-10 | **0.95** | Snake fall | **yes** |
| 32324 | 10J7756 | Fall | 06-May-08 | Snake, Hells Canyon Dam | Oxbow H | Snake R | IDFG | 03-Aug-10 | **0.99** | Snake fall | **yes** |
| 33463 | 10J3315 | Fall | 07-Apr-08 | Snake R L | Lyons Ferry H | Snake R L | WDFW | 18-Jul-10 | **0.97** | Snake fall | **yes** |
| 29016 | 10J7711 | Fall | 07-Apr-08 | Snake R L | Lyons Ferry H | Snake R L | WDFW | 27-Jul-10 | **0.91** | Snake fall | **yes** |
| 32199 | 10J2857 | Summer | 16-Apr-08 | Simukameen R |  | Methow & Okanogan | WDFW | 06-Jun-10 | **0.99** | U Columbia su fall | **yes** |
| 30546 | 10J3320 | Fall | 05-Mar-08 | Umatilla R | Umatilla H | Kalama R | ODFW | 27-Jul-10 | **0.95** | U Columbia su fall | **yes** |
| 35442 | 10J6115 | Summer | 12-May-06 | Columbia R, general |  | Wells H | WDFW | 27-May-10 | **1.00** | U Columbia su fall | **yes** |
| 18325 | 10J3197 | Summer | 12-May-07 | Chelan R + Columbia |  | Wells H | WDFW | 06-Jun-10 | **1.00** | U Columbia su fall | **yes** |
| 18472 | 10J3194 | Summer | 06-Apr-08 | Columbia R, general |  | Wells H | WDFW | 08-Jun-10 | **0.99** | U Columbia su fall | **yes** |
| 35347 | 10J5538 | Spring | 16-Sep-08 | Rogue R | Cole Rivers H | Cole Rivers H | ODFW | 26-Jun-10 | **0.98** | Rogue | **yes** |
| 33376 | 10J3234 | Spring | 18-Jul-07 | Trask R | Trask R H | Trask R H | ODFW | 20-Jun-10 | **0.99** | Mid Oregon Coast | **no** |
| Barcode | Coded-wire-tag ID | Chinook Run | CWT Release Date | Release Site | Hatchery | Stock | Release Agency | CWT Recovery Date | **GSI Posterior Probabilty** | GSI Stock assignment | GSI Correct? |
|  |  |  |  |  |  |  |  |  |  |  |  |
| 6143 | 10J0001 | URB L-Fall | 26-May-08 | Big Canyon Accl Pond | Lyons Ferry H | Lyons Ferry H | NEZP | 08-Jun-10 | **0.91** | N Puget Sound | **no** |
| 30539 | 10J3322 | Fall | 07-Apr-08 | Snake R L | Lyons Ferry H | Snake R L | WDFW | 27-Jul-10 | **0.99** | Deschutes fall | **no** |
| 35323 | 10J2901 | URB L-Fall | 01-Jul-08 | Youngs R & Bay | CEDC Youngs Bay Net | Cole Rivers H | ODFW | 12-Jun-10 | 0.44 | L Columbia fall | n/a, no, out of basin transfer |
| 18389 | 10J3228 | Late Fall | 02-Jan-08 | Coleman NFH | Coleman NFH | Coleman NFH | FWS | 19-Jun-10 | 0.65 | U Columbia su fall | n/a (no) |
| 35322 | 10J2902 | Fall | 07-May-08 | San Pablo Bay Net Pen | Feather R H | Feather R | CDFG | 12-Jun-10 | 0.81 | Klamath | n/a (no) |
| 33351 | 10J2999 | Spring | 01-Mar-07 | Gobar CR | Gobar Pond | Kalama R | WDFW | 29-Jun-10 | 0.70 | L Columbia fall | n/a (no) |
| 29109 | 10J3350 | URB L-Fall | 14-Apr-08 | Captain John | Lyons Ferry H | Lyons Ferry H | NEZP | 04-Aug-10 | 0.79 | Mid Oregon Coast | n/a (no) |
| 7682 | 10J7713 | URB L-Fall | 10-Jun-08 | NEZP | NPT H | Lyons Ferry H | NEZP | 26-Jul-10 | 0.87 | Mid Oregon Coast | n/a (no) |
| 18178 | 10J7723 | URB L-Fall | 14-Apr-08 | Captain John | Lyons Ferry H | Lyons Ferry H | NEZP | 25-Jul-10 | 0.86 | Snake fall | n/a (yes) |
| 32276 | 10J3321 | Fall | 07-Apr-08 | Snake R L | Lyons Ferry H | Snake R L | WDFW | 26-Jul-10 | 0.73 | Central Valley fall | n/a (no) |
| 32011 | 10J2938 | Fall | 07-Apr-08 | Snake R L | Lyons Ferry H | Snake R L | WDFW | 23-Jun-10 | 0.79 | Snake fall | n/a (yes) |
| 33377 | 10J3235 | Fall | 02-Jun-08 | Snake R L | Lyons Ferry H | Snake R L | WDFW | 20-Jun-10 | 0.80 | U Columbia su fall | n/a (no) |
| 7687 | 10J7710 | Fall | 10-Apr-08 | Spring CR | Spring CR NFH | Spring CR | FWS | 26-Jul-10 | 0.85 | L Columbia fall | n/a (no) |
| 18387 | 10J3227 | Fall | 10-Apr-08 | Spring CR | Spring CR NFH | Spring CR | FWS | 19-Jun-10 | 0.58 | Mid Columbia tule | n/a (yes) |
| 35249 | 10J6136 | Fall | 10-Apr-08 | Spring CR | Spring CR NFH | Spring CR | FWS | 27-Jun-10 | 0.70 | Mid Columbia tule | n/a (yes) |
| 35247 | 10J6135 | Summer | 21-Apr-08 | Wenatchee R |  | Wenatchee R | WDFW | 27-Jun-10 | 0.73 | Mid Oregon Coast | n/a (no) |

Abbreviations: Accl = Acclimation; CDFG = California Department of Fish and Game; COR = Columbia River; CR = creek; FWS = United States Fisheries and Wildlife Service; H = Hatchery; INS=Installation; L = Lower ; NEZP = Nez Perce Tribal H; NFH= National Fish Hatchery; ODFW = Oregon Department of Fish and Wildlife; R = River; S= south; su = summer; U = upper; URB = upriver bright; WDFW = Washington Department of Fish and Wildlife;
